# Redox signalling regulates breast cancer metastasis via phenotypic and metabolic reprogramming due to p63 activation by HIF1α

**DOI:** 10.1038/s41416-023-02522-5

**Published:** 2024-01-18

**Authors:** Zuen Ren, Malindrie Dharmaratne, Huizhi Liang, Outhiriaradjou Benard, Miriam Morales-Gallego, Kimita Suyama, Viney Kumar, Atefeh Taherian Fard, Ameya S. Kulkarni, Michael Prystowsky, Jessica C. Mar, Larry Norton, Rachel B. Hazan

**Affiliations:** 1 Department of Pathology, Albert Einstein College of Medicine, Bronx, NY 10461 USA; 2 Center for Cancer Research, Massachusetts General Hospital and Harvard Medical School, Boston, MA 02114 USA; 3 Australian Institute for Bioengineering and Nanotechnology, University of Queensland, Brisbane, QLD Australia; 4 Francisco de Vitoria University, Madrid, Spain; 5 Department of Endocrinology, Albert Einstein College of Medicine, Bronx, NY 10461 USA; 6 Department of Medicine, Memorial Sloan-Kettering Cancer Center, New York, NY 10021 USA

**Keywords:** Breast cancer, Mechanisms of disease

## Abstract

**Background:**

Redox signaling caused by knockdown (KD) of Glutathione Peroxidase 2 (GPx2) in the PyMT mammary tumour model promotes metastasis via phenotypic and metabolic reprogramming. However, the tumour cell subpopulations and transcriptional regulators governing these processes remained unknown.

**Methods:**

We used single-cell transcriptomics to decipher the tumour cell subpopulations stimulated by GPx2 KD in the PyMT mammary tumour and paired pulmonary metastases. We analyzed the EMT spectrum across the various tumour cell clusters using pseudotime trajectory analysis and elucidated the transcriptional and metabolic regulation of the hybrid EMT state.

**Results:**

Integration of single-cell transcriptomics between the PyMT/GPx2 KD primary tumour and paired lung metastases unraveled a basal/mesenchymal-like cluster and several luminal-like clusters spanning an EMT spectrum. Interestingly, the luminal clusters at the primary tumour gained mesenchymal gene expression, resulting in epithelial/mesenchymal subpopulations fueled by oxidative phosphorylation (OXPHOS) and glycolysis. By contrast, at distant metastasis, the basal/mesenchymal-like cluster gained luminal and mesenchymal gene expression, resulting in a hybrid subpopulation using OXPHOS, supporting adaptive plasticity. Furthermore, p63 was dramatically upregulated in all hybrid clusters, implying a role in regulating partial EMT and MET at primary and distant sites, respectively. Importantly, these effects were reversed by HIF1α loss or GPx2 gain of function, resulting in metastasis suppression.

**Conclusions:**

Collectively, these results underscored a dramatic effect of redox signaling on p63 activation by HIF1α, underlying phenotypic and metabolic plasticity leading to mammary tumour metastasis.

## Background

Metastasis is a systemic disease that is often incurable with currently available therapies [1]. It is believed that tumour heterogeneity drives metastasis by selecting for tumour cell subpopulations that are endowed with EMT and stemness properties [2]. Cancer cell hyper-proliferation leads to cell crowding, resulting in depletion of nutrients and oxygen, causing hypoxia. Tumours primarily use glucose metabolism or the Warburg effect to build energy and biomass [3]; however, emerging evidence has demonstrated that aggressive cancer cells use the TCA cycle and OXPHOS [4–6]. This in turn generates reactive oxygen species (ROS), which stabilise HIF1α, thereby driving cell proliferation, migration, survival, metabolism, and importantly EMT, which causes stemness, chemoresistance, and metastasis [7]. HIF1α stimulates EMT by activating e.g. *Snai1/2* and *Zeb1* gene expression, resulting in epithelial gene repression, involving *Cdh1* (E-cadherin) among other epithelial genes [8].

Our recent work highlights the profound effects of redox signalling by GPx2 KD on the mammary tumour phenotype involving ROS/HIF1α signalling, causing vascular malfunction, resulting in hypoxia and metabolic plasticity [9, 10]. In fact, GPx2 downregulation is a physiologically relevant change in breast cancer; analysis of a database of 1,809 patients, showed an association of GPx2 mRNA downregulation in luminal B, HER2-enriched, and basal-like tumours with poor patient survival [9]. Examination of similarly-sized control PyMT and PyMT/GPx2 KD tumours confirmed that GPx2 KD actively promotes metastasis, thus ruling out hypoxia caused by tumour bulkiness as the impetus for advancing malignancy [9]. However, the mechanism of metastatic progression by GPx2 KD remained unclear. EMT is a pivotal process which transforms epithelial carcinoma cells into mesenchymal-like cells, resulting in gain of stemness and metastatic traits [11]. However, recent studies have shifted our view on EMT, indicating a dynamic rather than a binary process, giving rise to partial epithelial to mesenchymal transitions spanning an EMT continuum, thereby potentiating tumour heterogeneity [12–16]. Interestingly, hybrid EMT subpopulations were observed in basal-like breast cancer cells, and were shown to exhibit tumour initiating and metastatic potential relative to cells residing at either end of the EMT spectrum [13–15, 17]. In light of these findings, we sought to investigate whether GPx2 KD promotes EMT dynamics at the single cell level at the primary tumour and distant sites to unravel unique subpopulations and/or oncogenic drivers governing metastasis.

Using scRNA-seq of the PyMT/GPx2 KD vs PyMT control tumour model, we identified six overlapping luminal clusters and one basal/mesenchymal-like cluster (cluster 3) enriched in EMT genes. Interestingly, GPx2KD tumour cell populations gained mesenchymal gene expression, resulting in hybrid epithelial/mesenchymal (E/M) clusters and a highly mesenchymal cluster (cluster 3). ScRNA-seq of integrated transcriptomics between lung metastases (mets) and primary GPx2KD tumour unravelled four luminal clusters and one basal/mesenchymal-like cluster (cluster 2). Interestingly, lung mets clusters gained basal/mesenchymal and luminal gene expression relative to primary tumour, thereby generating hybrid clusters, especially cluster 2. Importantly, E/M cells at the primary tumour expressed phosphorylated-AMPK and GLUT1, indicative of OXPHOS and glycolysis, whereas clusters in lung mets, notoriously cluster 2, used OXPHOS, underscoring the metabolic plasticity of the hybrid state. Furthermore, GPx2KD/HIF1α caused dramatic upregulation of p63 in hybrid tumour cell populations at the primary tumour and lung mets. Importantly, these effects were reversed by HIF1α inhibition or GPx2 overexpression, resulting in metastasis suppression. In sum, our findings underscore the role of GPx2KD/HIF1α/p63 signalling in regulating partial EMT and MET, underlying metabolic plasticity and metastasis.

## Methods

### Cell lines

Primary mammary tumour cell lines (PyMT1 and PyMT2) were generated by our laboratory from PyMT transgenic mouse tumours as described [9]. Briefly, tumours were excised, washed in PBS and diced with a razor blade into small fragments. Each gram of tumour was incubated with 10 ml of digestion media (1 mg/ml Collagenase type I, 100 unit/ml Hyaluronidase, 100 units/ml Penicillin/streptomycin, 2 mg/ml of BSA in Medium 199). The suspension was incubated at 37 °C for 3 h with occasional mixing. Digested material was spun at 1000 rpm for 5 min and pelleted cells were plated overnight at high density (1 × 10^7^ cells per 10 cm dish) in DMEM/20%FBS supplemented with 10 μg/ml insulin and 20 μg/ml EGF. Fibroblasts were depleted by limiting trypsinisation. Epithelial cells were grown for several weeks till they reached crisis and adapted to in vitro culture conditions. The human JIMT1 BC cell line was cultured in DMEM medium supplemented with 10% FBS and 1% Pen-Strep.

### Animal studies

Mice were housed and maintained by the Animal Studies Institute at the Albert Einstein College of Medicine. Animal protocols were reviewed and approved by the Institute for Animal Studies. MMTV-PyMT transgenic mice [18] producing mammary tumors, were used to isolate  cell lines that were  non-metastatic (PyMT1) or highly metastatic (PyMT2) to the lungs. One of each of these cell lines  was used to knockdown or overexpress GPx2 respectively as described [9]. PyMT1, PyMT2, or JIMT1 control and modified cell lines (1 million cells suspended in 200 μl PBS) were each injected bilaterally into m.f.p. of athymic female nude mice (Jackson laboratories) to generate mammary xenografts.

### Tumour growth kinetics

Tumour growth curve was determined by measuring tumour volume twice a week using a caliper. Tumour volume was determined upon the formula: tumour volume = shorter diameter^2^ × longer diameter/2. Mice (three per group for PyMT2/dCas vs PyMT2/dCas-GPx2-gRNA2; ten per group for PyMT1/GPx2 KD treated with vehicle vs echinomycin; three per group for JIMT1 control vs JIMT1/GPx2 OE) were sacrificed at end point. Data are displayed as mean tumour volume ± SEM. Statistical analyses were performed comparing individual time points by unpaired *t* test and significant differences were established as *p* value < 0.05.

### Lung metastasis

Mice bearing mammary tumours were sacrificed at end point of 1–2 cm diameter *per* our animal protocol. Lungs were inflated by tracheal cannulation with injection of 1–2 ml of 10% neutral buffered formalin. Formalin-fixed lungs were paraffin-embedded and blocks sectioned on a tissue microtome (Leica Microsystems) at 5 μm. Lungs were serial sectioned through the tissue as sets of 5 serial sections, at 300-μm intervals. Analysis was performed on whole sections after it was determined by inspection that metastases seeded in random locations. Data are displayed as mean foci number ± SEM. Statistical analyses used unpaired t-test and significance was determined at *p* < 0.05.

### GPx2 expression constructs

The open reading frame of mouse GPx2 (GenScript NM_030677.2) was subcloned by PCR into Xho1/BamH1 restriction sites of lentiviral expression vector pLVX-puro (Clontech), using 5’ region primer: 5’-TAT CTC GAG GCC ACC ATG GCT TAC ATT GCC AAG TCG-3’ and 3’ region primer: 5’-TAT GGA TCC CTA GAT GGC AAC TTT GAG GAG CCG-3’, and the open reading frame of human GPx2 (GenScript NM_002083.4) was subcloned by PCR into Xho1/BamH1 restriction sites of lentiviral expression vector pLVX-puro (Clontech), using 5’ region primer: 5’-CCC CTC GAG ATG GCT TTC ATT GCC AAG TCC TTC TAT GAC-3’ and 3’ region primer: 5’-CCC GGA TCC CTA TAT GGC AAC TTT AAG GAG GCG CTT GAT-3’. Recombinant lentiviruses expressing mGPx2 and hGPx2 were packaged in 293 T cells and expressed in PyMT2 and JIMT1 respectively as described below.

The third generation lentiviral transfer plasmid pXPR_dCas9-VPR_sgRNA was cloned from pXPR_dCas9-VP64-Blast (Addgene plasmid #61425) [19]. For mouse GPx2 sgRNA design, two custom anti-sense DNA oligonucleotides (CTTTGTTCAGTGGCAGTAAG, TTGTTCAAACAGTTCACAGG) were annealed and ligated into pXPR_dCas9-VPR_sgRNA. Non-targeting pXPR_dCas9-VPR with no GPx2-sgRNA inserted recognition sequence was used as a control. Lentiviral construct for dCas9-VPR-sgRNA and virus particles was prepared by Memorial Sloan Kettering Cancer Center, New York.

### GPx2 shRNA-mediated knockdown

Lentiviral mission shRNA clones against mouse GPx2 (TRCN0000076528, TRCN0000076529, TRCN0000076530, TRCN0000076531, and TRCN0000076532) were purchased from Sigma. ShRNA lentiviral vectors against mouse GPx2 and a control non-targeting shRNA were packaged in 293 T cells and infected into cells. Briefly, PyMT1 cells were seeded at of 1 × 10^5^ per 12-well plate for 24 h, treated with 250 μl viral solution containing 10 μg/ml polybrene for 1 h and incubated in DMEM/10 % FBS without antibiotics for 24 h. Cells were expanded into a 10-cm dish with selective antibiotics.

### Lentivirus production and transduction

Lentiviral particles were generated by transient co-transfection of 293 T cells with lentivirus-based vector expressing either the full-length clone of desired gene or an shRNA sequence to target-specific RNA of gene. Briefly, a 100-mm dish seeded with 3 × 10^6^ cells were transfected with 0.6 μg of lentiviral packaging gene TAT, 102 RVE, and GAG/POL and 1.2 μg of VSV-G and 12 μg of DNA of interest in lentiviral backbone. Fugene was used to transfect the cells. Following 48 h of transfection, supernatant was collected, centrifuged at 2000 rpm for 10 min, and filter sterilised. Cells were seeded at 1 × 10^5^ per 12-well plate for 24 h, treated with 250 μl viral solution containing 10 μg/ml polybrene for 1 h and incubated in DMEM/10% FBS without antibiotics for 24 h. Cells were expanded into a 10-cm dish with selective antibiotics.

### Antibodies and reagents

Antibodies against GPx2 (AB137431), HIF1α (AB228649), NOTCH1 (AB52627), pAMPK (staining, AB23875) were obtained from Abcam. Rabbit polyclonal anti TWIST (sc-15393), was obtained from Santa Cruz Biotechnology. Rat anti KRT8 (staining, Cat# TROMA-I; RRID: AB_531826) was purchased from DSHB. Rabbit anti p63 (staining, 39692), VIM (staining and WB, 5741), SLUG (9585), E-CAD (staining and WB, 3195), pAMPK(WB, 2535), and mouse anti SNAI1 (3895) were from Cell Signaling and β-actin antibody was from Sigma. Rabbit anti KRT14 antibody (staining, 905304) was from Biolegend. Guinea pig anti KRT14 (staining, Cat#. GP-CK14) was from Progen. Mouse anti E-cad (WB and staining, 610182) and mouse anti N-cad (staining, 610920) was from BD Transduction Labs. Rabbit anti-SLC2A1(GLUT1) antibody (WB, MBS9126610) was purchased from BioSource. Mouse anti-SLC2A1(GLUT1) (staining, NBP-75785-100ug) was purchased from Novus Biologicals. Alexa Fluor 405, 488, 594 goat anti mouse or rabbit or rat, Alexa Fluor 647 donkey anti guinea pig were from Invitrogen. Echinomycin was obtained from Tocris Bioscience.

### Immunoblotting

Cells or tissues were extracted in RIPA solubilisation buffer (50 mM Tris-HCL pH 7.5), 150 mM NaCl, 0.5 mM MgCl2, 0.2 mM EGTA, 1% Triton X-100) including protease and phosphatase inhibitors. 30 μg protein were loaded on 7–12% SDS-polyacrylamide gels and transferred to Immobilon membranes. Blots were probed overnight at 4 °C with indicated antibodies and developed by chemiluminescence (Perkin Elmer).

### Immunofluorescence

Formalin-fixed/paraffin-embedded tumour tissues were sectioned in 5 μm thickness, deparaffinised in xylene, and rehydrated in a series of 100% ethanol, 95% ethanol, and distilled water. Antigen retrieval was performed 1× antigen retriever solution (Sigma, pH 6.0). Tissues were incubated with primary antibody in 5% donkey serum, 2% BSA, 0.5%TX-100 in TBS, followed by incubation of Alexa Fluor secondary antibody for 1.5 h at room temperature, washed and counterstained with DAPI to visualise nuclei.

### ROS measurements

ROS detection reagent, 2′,7′-dichlorofluorescein (DCF) (Invitrogen, C6827) was used to determine intracellular ROS levels. DCF was reconstituted using anhydrous dimethylsulfoxide (DMSO, Sigma) with optimal working concentration at 10 μM in DMEM/2% serum. Media with DCF were added to cells in 96 wells and incubated for 1 h in the dark at a 37 °C, 5% CO_2_ incubator. After incubation, media with DCF was removed and cells were washed twice with PBS. DCF fluorescence intensity relative to background fluorescence in wells that do not contain cells was determined using a fluorescent microplate reader using excitation wavelength at 495 nm and emission wavelength at 520 nm. The results were normalised to cells/well. Data are displayed as Mean H2DCFDA fluorescence intensity ± SEM.

### Oxygen consumption in vitro

Cells were suspended in normal growth medium at a concentration of 1 × 10^5^ cells/ml, and 100 μl of cells was seeded on Seahorse 96-well plates 48 h prior to performing the assay. Oxygen consumption rate (OCR) assay was carried out in a XF96 Seahorse Analyzer (Seahorse Bioscience, Billerica, MA, USA). On the day of the assay, medium was replaced with prewarmed (37 °C) 180 μl Seahorse medium (Seahorse XF medium with 2 mM glutamine, 10 mM glucose, 1 mM pyruvate) and the plate was incubated for 1 h in a 37 °C non-CO2 incubator. The mitostress test was performed with 1 μM oligomycin, 1 μM FCCP, and 0.5 μM rotenone/antimycin. The final OCR values were normalised to cells/well using the CyQUANT Cell Proliferation Assay Kit (Thermofisher).

### Glycolysis stress test in vitro

Cells were suspended in normal growth medium at a concentration of 1 × 10^4^ cells/ml, and 100 μl of cells was seeded on Seahorse 96-well plates 48 h prior to performing the assay. Glycolysis stress assay was carried out in a XF96 Seahorse Analyzer (Seahorse Bioscience, Billerica, MA, USA). On the day of the assay, medium was replaced with prewarmed (37 °C) 180 μl of XF Base Medium (Agilent Technologies, 103335) supplemented with 2 mM glutamine and incubated for 1 h at 37 °C in a non-CO_2_ incubator. Baseline extracellular acidification rate (ECAR) measurements were recorded 4 times (mix: 3 min; wait: 2 min; measure: 3 min), followed by sequential injection of 20 mM glucose, 1 μM oligomycin, and 50 mM 2-DG with 4 readings (mix: 3 min; wait: 2 min; measure: 3 min) after each injection. The ECAR values were normalised to cells/well using the CyQUANT Cell Proliferation Assay Kit (Invitrogen, C7026).

### Cell isolation of mammary tumour cells for single-cell RNA sequencing

PyMT1 control, PyMT1/GPx2 KD tumour, and lung metastases (from paired PyMT1/GPx2 KD primary tumour) generated by injection of GFP-labelled tumour cell lines, was excised and mechanically chopped with scalpels, incubated in DMEM/F12 with 5% FBS, 5 μg/ml insulin, 2 mg/ml collagenase, and 150 μg/ml DNase at 37 °C for 45 min. Cell clamps were washed and dissociated with 2 ml of 0.05% trypsin/EDTA at 37 °C for 10 min and trypsinisation stopped with 10 ml PBS–2%FBS-EDTA. The samples were filtered through a 70 μm cell strainer, pelleted at 1200 rpm at 4 °C for 5 min, and resuspended in 1 ml of 1× red blood cell (RBC) lysis buffer and 10 ml PBS-2%FBS-EDTA was added to stop lysis. Cell pellets were resuspended in 1 ml PBS–2%FBS-EDTA and viability-controlled by trypan blue dye exclusion.

### Flow cytometry

GFP-labelled cells from PyMT1 control, PyMT1/GPx2 KD tumour, and lung metastases were sorted using a FACSAria II (BD Biosciences). Singlets were selected by using standard forward scatter width vs area. Cell viability was controlled by DAPI staining and single-cell suspensions were sorted in DMEM/10% FBS.

### Library preparation and single-cell RNA sequencing

Library preparation was performed by Mr. David Reynolds at the AECOM genomic core facility. GFP-sorted single-cell suspensions from PyMT1 control, PyMT1/GPx2 KD tumour, and lung metastases containing at least 80% viable cells were processed to capture viable cells. The libraries were prepared (individual lanes on the 10× Chromium) using the 10× Single Cell 3’ v3 kit using ~10,000 cells per lane on the 10× Chromium microfluidics device (10× Genomics, Pleasanton, CA). The qualified libraries were sequenced on Illumina Hiseq 6000 platform (Novogene Corp, Sacramento, CA), with a standard paired-end 150 bp (PE150) at each end.

### Processing of scRNA-seq data

The original data obtained from Illumina® HiSeq platform (Novogene Corp, Sacramento, CA), were transformed to sequenced reads by base calling. Raw data obtained as FASTQ files which contain sequenced reads were subjected to quality control using OpenGene’s fastp v0.19.4 preprocessor [20]. Cellranger 3.0.2. software was used to provide reads alignment and gene annotation to the mm10 mouse reference genome. The STAR aligner employed within cellranger 3.0.2 was used to perform alignment. Fragments Per Kilobase of transcript per Million mapped reads (FPKM) values were quantified using each cell-barcode in combination with unique molecular identifiers (UMIs) using the default cellranger. The quantified UMI counts were used to form an unfiltered gene-barcode matrix and exported for further downstream analysis.

### Quality control and normalisation

The unfiltered gene-barcode matrix for each sample was imported into R 3.6.1 and converted into SingleCellExperiment (SCE) objects. Any barcodes with a total UMI count greater than 200 were classified as cells. Seurat 3.1.1 was used for further quality control and preprocessing of the exported SCE objects. Cells with a minimum cut-off of 200 and a maximum cut-off of 4500 total RNA count were filtered for primary tumour single-cell data; cells with a minimum cut-off of 200 and a maximum cut-off of 7000 total RNA count were filtered for lung met single cell data. In addition, cells with a percentage of total reads that aligned to mitochondrial genome greater than 10% were removed (inferred as cells undergoing stress and cell death). After quality control, a total of 5466 cells from PyMT1 control tumour, 5772 cells from PyMT1/GPx2 KD tumour, and 709 cells from lung metastases were used for normalisation and downstream analyses. Cells passing QC were normalised using the SCTransform pipeline in Seurat 3.1.1.

### Integration and clustering

After normalisation, the data obtained from PyMT1 control, PyMT1/GPx2 KD tumour, and lung metastases underwent comprehensive integration using the integration pipeline in Seurat with the genes commonly retained between control and GPx2 KD tumour, GPx2 KD tumour and lung metastases serving as integration anchors [21]. Integration refers to the process of combining datasets while taking into account sources of variation that may come from different technical or batch effects. Principal component analysis was performed to enable unsupervised clustering on the integrated data from PyMT1 control and PyMT1/GPx2 KD tumour, and PyMT1/GPx2 KD tumour and its lung metastases. Clustered cells were projected in two dimension using Uniform Manifold Approximation and Projection (UMAP) for cluster visualisation [22] Louvain community detection algorithm was further applied to identify the cluster partitions, with a resolution parameter 0.4 that were identified by building a clustering tree on the datasets to optimise the number of clusters.

### Cluster identification and annotation

For cluster identification in the integrated dataset, we performed manually supervised-automated methods of cell-type identification. Canonical cell-type marker genes for basal (e.g. *Krt5, Krt14, Krt17*) and luminal cells (e.g. *Krt8, Krt18, Cldn*) were annotated to identify which clusters belonged to which cell type.

### Cluster comparison and visualisation

To examine cluster specific genes, we used feature plot functions to highlight expression patterns of marker genes of interest. Violin plots were used to visualise the expression pattern of genes of interest across cell clusters.

### Cell trajectory analysis

Pseudotime cell trajectory analysis was performed using Monocle 3 package (https://cole-trapnell-lab.github.io/monocle3). The batch corrected data between control and GPx2 KD tumour, GPx2 KD and paired lung mets, were integrated using Seurat and were used as input to Monocle 3. The top 2000 highly variable genes in the integrated dataset were used to compute the pseudotime ordering and the cell trajectory analysis. Cells were plotted in UMAP space for visualisation of pseudotime trajectories after clustering. We examined the expression of some of the luminal/epithelial and basal/mesenchymal genes found to be differentially expressed across the pseudotime trajectory.

### Differential gene expression analysis

To investigate gene expression change by cluster comparison, a joint variable of condition × cell type was used to calculate cluster-type-specific differential expression. Genes with an adjusted *p* value <0.05 were classified as significantly differentially expressed. The differential gene expression analysis was carried out using a Wilcoxon Rank sum test with Benjamini–Hochberg test for multiple testing correction, with the default parameters of the *FindMarkers()* function in Seurat.

### Pathway overrepresentation analysis

Overrepresentation pathway analysis was performed on the differentially expressed genes between each condition by using KEGG pathway database. Pathways with an FDR-adjusted *p* value <0.1 were considered to be significantly overrepresented. Mouse MSigDB Hallmark gene sets were downloaded from the Walter and Eliza Hall Institute of Medical Research webpage (http://bioinf.wehi.edu.au/software/MSigDB/). Mouse gene symbols were converted to their Entrez ID. Gene set enrichment analysis was carried out on the voom normalised gene expression data.

### Gene set enrichment analysis

Gene set enrichment analysis (GSEA) was performed on the differentially expressed genes between each condition using *GSEA* function in clusterProfiler [23] R package. The input to the *GSEA* function is an order ranked gene list by the average log fold change between each condition. The Hallmark gene sets available via the msigdbr (version 7.4.1) R package were used as a priori defined set of genes for performing GSEA.

### Statistical analysis

Results represent the mean ± SEM (standard error of the mean) for indicated experiments with at least three independent biological replicas. The statistical methods used are described in the figure legends. Most data were analysed by the unpaired two-tailed Student’s *t* test to compare two groups with significance set at the *p* value < 0.05. Statistical analysis was performed using the Prism 9 (GraphPad) software.

## Results

### The identification of a basal/mesenchymal-like subpopulation in primary tumour at the single cell resolution

**Fig. 1:**
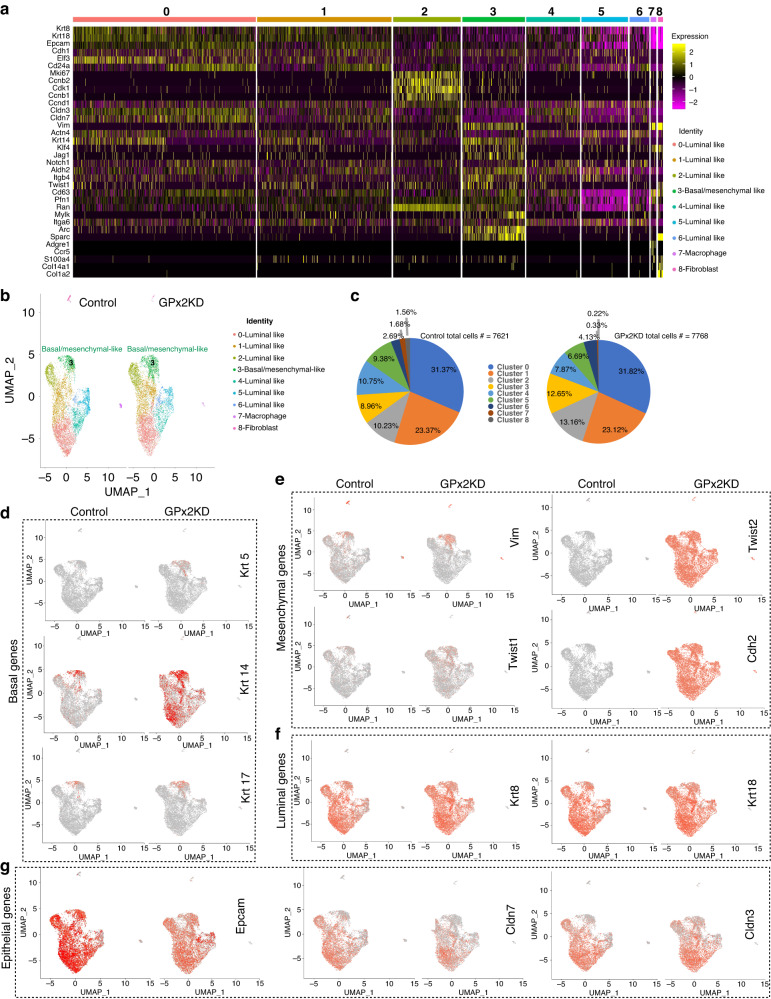
GPx2 KD stimulates mesenchymal gene expression in M-cluster 3 and luminal clusters. **a** Heatmap shows manually supervised-automated clustering of individually selected marker genes from integrated PyMT1/GPx2KD and control PyMT1 single cell RNA datasets. **b** UMAP projection of comprehensively integrated clustering results from one PyMT1 control and one PyMT1/GPx2 KD tumour revealed six luminal-like clusters (cluster 0, 1, 2, 4, 5, 6), one basal/mesenchymal-like cluster (cluster 3), and two non-epithelial clusters (clusters 7 and 8). **c** Pie graphs display the percentage of cells in each cluster in GPx2 KD vs control tumour. Pearson’s Chi-square test, *p* value = 2.2e−16; two-sample test for equality of proportions with continuity correction, data c (983, 683) out of c(7768, 7621), *p* value = 1.03e−13. **d**–**g** Feature plots in low-dimensional space showing expression of basal genes (*Krt5*, *Krt14*, *Krt17*) (**d**), mesenchymal genes (*Vim*, *Twist1*, *Twist2*, *Cdh2*) (**e**), luminal genes (*Krt8 and Krt18)* (**f**), epithelial genes (*Epcam*, *Cldn3/7*) (**g**) in the GPx2 KD tumour relative to control tumour.

We showed that GPx2 KD in the PyMT model enhanced spontaneous metastasis [9]; however, the mechanism remained unknown. scRNA-seq of FACS-sorted GFP-labelled cells from one PyMT1/GPx2 KD and one PyMT1/control mammary tumour unravelled seven carcinoma cell subpopulations (clusters) that were shared by the GPx2 KD and control tumour [9]. Of these, six were luminal-like and one was basal/mesenchymal-like. Cluster identification was based on manually supervised annotation using MSigDB hallmark genes indicative of cell type (basal, luminal) or cell state (proliferation status) (Fig. 1a). Cluster 3 exhibited a classical EMT/stem-like gene signature involving upregulation of basal/mesenchymal genes (*Vim, Krt14, Klf4, Jag1, Notch1, Aldh2, Itgb4, Twist, Sparc, S100a4*), and downregulation of luminal/epithelial genes (*Krt8/18, Epcam, Cdh1, Cldn3/7*) relative to all clusters (Fig. 1a). Of note, the expression of *Klf4, Jag1, Notch1, Aldh2* in cluster 3 indicates a population with stemness properties. By contrast, luminal cluster 2 was enriched in a proliferative gene signature (*Mki67, Ccnb2, Cdk1, Ccnb1 and Ccnd1*) (Fig. 1a), which may be associated with cycling progenitors fuelling tumour growth. In addition, we identified two non-epithelial clusters (clusters 7 and 8) with macrophage and fibroblast like signatures, referring to tumour-associated stromal cells (Fig. 1a).

To confirm the clustering data obtained by manual annotation, we unbiasedly analysed the differential expressed genes between cluster 3 and the luminal clusters combined (cluster 0, 1, 2, 4, 5, 6). This showed striking upregulation of basal *Krt17* and mesenchymal (*Col1a1, Vcam1, Sparc, Col18a1, S100a6*) genes in cluster 3 (Fig. S1A). In-depth analysis of cluster 3 revealed upregulation of genes that were basal-like (*Krt14, Krt17)*, mesenchymal-like (*Vim, Pdgf, Sparc, Arc, Mest, S100a6, Vcam1, Col1a1, Col18a1*) and stem-like (*Foxa1, Twist1, Jag1, Klf4, Itgb4*) genes (Fig. S1B), that coincided with downregulation of luminal genes (*Krt8/18, Cldn3/7, CD24, Epcam*), indicative of a classical EMT signature (Fig. S1B).

### GPx2 KD promotes EMT dynamics at the primary tumour via mesenchymal gene expression

We sought to determine whether GPx2 KD potentiates EMT via cluster 3. Analysis of all clusters in the GPx2 KD vs control mammary tumour in UMAPs (Fig. 1b) unravelled a significant increase in the size (cell number) of cluster 3 from 683 (8.96%) in control to 983 (12.65%) in GPx2 KD tumour (Fig. 1c). Conversely, luminal clusters 4 and 5 were reduced from 10.75% and 9.38% in control to 7.87% and 6.69% in GPx2 KD tumour (Fig. 1c). Conversely, luminal cluster 2 was increased from 10.23% in control to 13.16% in GPx2 KD tumour, indicative of proliferative activity (Fig. 1c). Hence, variations in cluster size by GPx2KD may reflect shifts in cluster identity, due to gain in mesenchymal genes which convert cells in luminal clusters 4 or 5 into cluster 3-like mesenchymal cells. We thus examined the effect of GPx2 KD on basal, mesenchymal and luminal gene expression in all clusters. Feature plots showed that GPx2 KD increased the expression of basal (*Krt5, Krt14, Krt17*) and mesenchymal (*Vim,Twist1/2*) genes, especially in cluster 3 (Fig. 1d, e), while reducing *Epcam* in most clusters, and *Cldn7* especially in cluster 3 (Fig. 1g). Strikingly, GPx2 KD induced de novo expression of basal *Krt14*, mesenchymal *Twist2* and *Cdh2* genes (Fig. 1d, e) in luminal clusters expressing (*Epcam Cldn3/7*, *Krt8/18*) (Fig. 1f, g). Collectively, these data demonstrate that GPx2 KD promotes a mesenchymal-like cluster (cluster 3) and several hybrid EMT (E/M) clusters expressing luminal and mesenchymal genes.

### Cell trajectory analysis suggests that GPx2 KD promotes an EMT spectrum

To determine whether GPx2 KD promotes EMT across a continuum, we constructed a pseudotime trajectory model which is an unbiased computational method that defines cell trajectories across all clusters using Monocle 3 [24] and in this context, helps to elucidate the spectrum of mesenchymal and epithelial lineage states. The principle behind this type of analysis is to group cell clusters that have similar degrees of variation to understand how these states transition from one to another (see Methods). We found that the main trajectory was consistent with our pre-set clustering results (Fig. S2A, B). Of note, cell trajectory and UMAP clustering projections were clearly indicative of transitions between M and E states, underscoring a rapport between mesenchymal and luminal cell lineages (Fig. S2B). Cluster 3 was enriched in mesenchymal and stem-like genes and was therefore placed at the root of the trajectory as the transcriptional initiating region. Indeed, we found that cluster 3 gave rise to three differentiation routes linking various tumour cell subpopulations (Fig. S2B). The trajectory between mesenchymal and luminal clusters was examined by the rise and fall of canonical epithelial or mesenchymal gene markers across pseudotime distance between the clusters. For example, *Krt14*, a basal-like cell marker, was highly expressed in mesenchymal cluster 3 with greatest pseudotime distance from the utmost luminal cluster (cluster 0) (Fig. S2C). Interestingly, *Krt14* displayed a slightly variable pattern in transition state across the clusters, more specifically falling away from cluster 3, rising again in cluster 5, and declining to lowest level in cluster 0 (Fig. S2C), pointing to cluster 5 as an early hybrid-like cluster. By contrast, luminal *Krt18* or *Epcam* was lowest in cluster 3, slowly rose in luminal-like clusters and plateaued in cluster 0 (Fig. S2D, E). Conversely, the expression of mesenchymal *S100a6*, and to a lesser extent also of *Col1a1*, was highest in cluster 3, but gradually declined across the luminal-like clusters (Fig. S2F, G). These results suggested that GPx2 KD promotes intermediate E to M transitions, generating a continuum of EMT states, with cluster 3 and cluster 0 residing at opposite ends of the spectrum.

### GPx2 knockdown promotes hybrid EMT states in the primary tumour in vivo

**Fig. 2:**
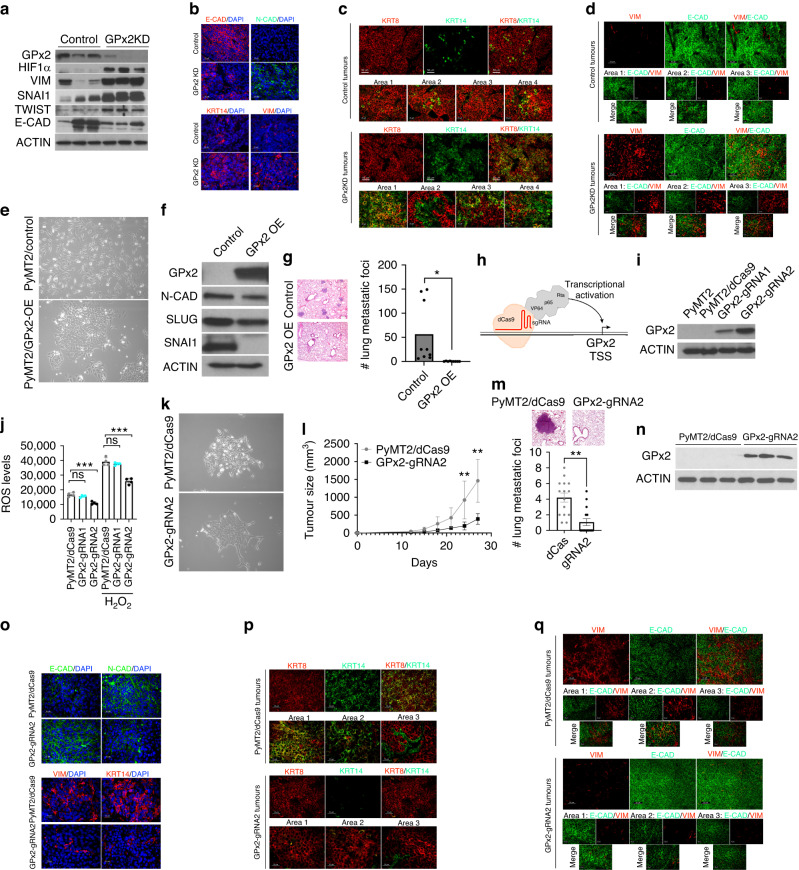
GPx2 KD promotes EMT dynamics in vivo that were reversed by GPx2 gain of function. **a** Western blots of PyMT1/GPx2 KD vs control PyMT1 tumour lysates (*n* = 3 mice), for expression of GPx2, HIF1α, VIM, SNAI1, TWIST, E-CAD relative to ACTIN. **b** Immunofluorescent (IF) staining for E-CAD, N-CAD, KRT14, VIM in GPx2 KD vs control tumours (*n* = 5 mice; 3 sections each). **c**, **d** IF staining for KRT8 and KRT14 (**c**), VIM and E-CAD (**d**) in control and GPx2 KD tumours (*n* = 5 mice); Representative images illustrate different EMT states in various tumour areas. (**E**) Phase-contrast images of PyMT2 cells and PyMT2/GPx2OE cells at ×10 magnification. **f** Western blots of PyMT2 and PyMT2/GPx2OE cell lysates for expression of GPx2, N-CAD, SLUG, SNAI1 vs ACTIN. **g** Bar graphs show the number of metastatic foci per lung section (*n* = 3) from 3 female athymic mice that were tail-vein injected with PyMT2 vs PyMT2/GPx2OE cells; Mean ± SEM; *p < 0.05. Images of lung foci are shown. **h** Schematic of the sgRNA-dCas9-VPR complex. Transactivators VP64, p65, and Rta were directly fused with C-terminal of dCas9. **i** Two GPx2 targeting sgRNAs-dCas9-VPR were used to endogenously activate GPx2 expression in PyMT2 cells, and dCas9-VPR without sgRNA was used as control. **j** ROS levels are shown as Mean ± SEM; ***p < 0.001, ns indicates non-significance. **k** Phase-contrast images of PyMT2/dCas9 control and PyMT2/GPx2-gRNA2 cells at 10x magnification. **l** Control and GPx2-gRNA2 cells (1 × 10^6^) were bilaterally injected into mammary fat pads of female athymic nude mice (*n* = 3 each group). Tumour growth curves over 28 days post tumour onset are shown as Mean of tumour volume ± SEM; **p < 0.01. **m** Scans of H&E stained sections of lung (boxes) from mice carrying control or GPx2-gRNA2 tumours (upper panels); Graph showing average number of foci per lung section (*n* = 5) from 3 mice per group; Mean ± SEM; ***p < 0.001. **n** IB shows GPx2 vs ACTIN in GPx2-gRNA2 vs control PyMT2 tumours (*n* = 3 mice). **o** IF images from E-CAD vs N-CAD (top panels) and KRT14 vs VIM (bottom panels) in GPx2-gRNA2 vs control tumours (*n* = 6). **p**, **q** Co-staining of KRT8/KRT14 (**p**) (top), VIM/E-CAD (**q**) (bottom) in GPx2-gRNA2 vs control tumours (*n* = 6); 3 mice each.

To validate these findings in vivo in the tumour context, we examined the relative expression of canonical epithelial and mesenchymal markers in PyMT1/GPx2KD vs control PyMT1 tumours. Immunoblotting of mammary tumour chunks showed dramatic upregulation of HIF1α that was concomitant with increased VIM, SNAI1, TWIST and decreased E-CAD expression in GPx2 KD relative to control tumours (Fig. 2a). Immunostaining revealed that GPx2 KD tumours contained carcinoma areas that were KRT14, N-CAD, VIM positive and E-CAD negative as compared to control tumours (Fig. 2b), indicative of binary EMT. However, in agreement with feature plots, GPx2 KD tumours harboured areas expressing both E and M markers involving KRT14 (basal) and KRT8 (luminal) relative to control tumours expressing only KRT8 (Fig. 2c; Areas 1, 3, 4). Interestingly, GPx2 KD tumours also harboured areas that were either KRT14 or KRT8 positive (Fig. 2c; Area 2). These data were also confirmed by using E-CAD and VIM as E/M markers. Immunostaining revealed areas in the GPx2KD tumour that were E-CAD^high^ and VIM^high^ (Area 3, but also Area 2) relative to control tumours which were E-CAD^high^ (Fig. 2d), as well as areas that were either E-CAD^high^ or VIM^high^ (Area 1) (Fig. 2d). Together, these data suggested that GPx2 KD promotes an EMT spectrum encompassing luminal, luminal/mesenchymal, and mesenchymal phenotypes.

### GPx2 gain of function suppresses EMT dynamics and metastasis

We showed that GPx2 overexpression (OE) in the metastatic PyMT2 cell line suppressed metastasis [9]. In vitro, GPx2 OE induced a shift from a mesenchymal to an epithelial morphology, with reduced N-CAD, SNAI1 and SLUG levels (Fig. 2e, f). Similarly, GPx2 OE tumours, showed high E-CAD and low N-CAD/KRT14/VIM expression relative to control PyMT2 tumours (Fig. S3A). Interestingly, PyMT2 tumours were enriched in KRT8/KRT14 or E-CAD/VIM hybrid areas as compared to GPx2 OE tumours which expressed either KRT8 or E-CAD (Fig. S3B, C), indicative of mesenchymal to epithelial transition (MET). Of note, elongated VIM^+^ cells in control tumours may be cancer-associated fibroblasts (Fig. S3C). MET induction by GPx2 OE was supported by dramatically reduced lung colonisation following tail vein injection of tumour cells into female athymic mice (Fig. 2g). These data were further substantiated by GPx2 endogenous induction into PyMT2 cells using the CRISPR activation system. Using guide RNAs (sgRNAs) to direct dCas9-VPR to the GPx2 proximal promoter for de-repression and dCas9-VPR without sgRNA as control (Fig. 2h), we found that sgRNA2 was the most effective in inducing GPx2 protein (Fig. 2i). Moreover, GPx2 induction lowered ROS levels and shifted PyMT2 cells from M to E morphology (Fig. 2j, k). In vivo, PyMT2/gRNA2 cells showed reduced tumourigenic and metastatic potential (Fig. 2l–n), consistent with a shift towards MET, as indicated by E-CAD^high^/N-CAD-VIM-KRT14^low^ expression (Fig. 2o). Indeed, PyMT2/gRNA2 tumours were luminal-like (KRT8+ or E-CAD+), as compared to control tumours which were E/M-like as shown by KRT8/KRT14 or E-CAD/VIM expression (Fig. 2p, q). These data underscored that GPx2 inhibits E/M and M states, thus causing epithelial differentiation leading to suppression of tumour growth and metastasis.

### scRNA-seq of lung metastases reveals cluster homology with the primary GPx2 KD tumour

**Fig. 3:**
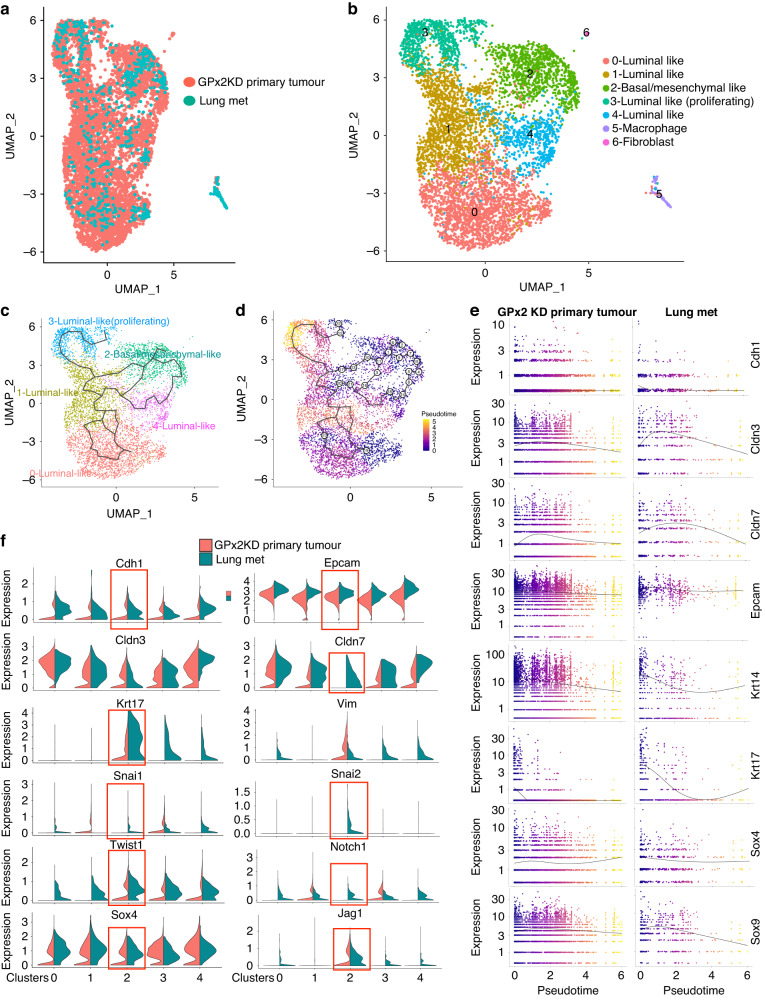
Pseudotime trajectory analysis illustrates the transcriptional EMT/MET continuum between clusters from primary GPx2 KD tumour and paired lung metastases. **a**, **b** UMAP projection of comprehensively integrated clustering data from GPx2 KD primary tumour and paired lung mets using the integration pipeline in Seurat, unravelled 7 clusters involving four luminal-like (cluster 0, 1, 3, 4), one basal/mesenchymal-like (cluster 2); one macrophage-like (cluster 5) and one fibroblast-like (cluster 6). **c**, **d** Pseudotime time trajectories (**d**) of integrated clustering projected onto UMAP (**c**); the bifurcating line illustrates a branched trajectory from the root of one of the terminal nodes, which was arbitrarily set as cluster 2 or the most mesenchymal transcriptional node; colour changes (**d**) reflect pseudotime distance between clusters (basal/mesenchymal cluster 2 located at *t* = 0). **e** Gene expression levels of epithelial/luminal genes (*Cdh1*, *Cldn3/7*, *Epcam*), basal/mesenchymal (*Krt14, Krt17)* and stemness (*Sox4, Sox9*) across pseudotime distance between the clusters in primary GPx2 KD tumour vs lung mets are shown as curves; colours indicate individual clusters in UMAP projection over pseudotime distance. **f** Violin plots show relative expression of genes that were either epithelial/luminal (*Cdh1, Epcam, Cldn3/7)*, basal/mesenchymal (*Krt17*,*Vim, Snai1/2,Twist1*), or stem-like (*Notch1, Jag1, Sox4*) in clusters from lung mets vs primary GPx2KD tumour, pointing to lung mets cluster 2 as the most hybrid (square).

To identify the subpopulation(s) driving spontaneous metastasis, we performed scRNA-seq of lung metastases (mets) that were derived from the parent PyMT1/GPx2 KD mammary tumour. To study the relationship between lung mets and primary GPx2 KD tumour, we used Seurat [16] to integrate the transcriptomics of both sites, as shown in UMAP (Fig. 3a, b). Integration refers to the process of combining datasets, while taking into account sources of variation that may come from different technical or batch effects, thus allowing for more meaningful comparisons. The merged transcriptomics unravelled four clusters (cluster 0, 1, 3, 4) expressing luminal genes (*Cldn3, Krt8, and Krt18*) (Fig. S4). Similar to luminal cluster 2 in primary tumour, cluster 3 in lung mets, was enriched in a proliferation gene signature (*Ran, Cks1b, and Cks2*) (Fig. S4), whereas cluster 2 (homologous to cluster 3 in primary tumour), expressed a mesenchymal-like gene set (*Col18a1, Vcam1, Col1a1, Vim, Sparc*) (Fig. S4). Finally, lung mets contained a macrophage-like (cluster 5), and a fibroblast-like (cluster 6) cluster, as indicated by corresponding Ccr5/Col4a1 and Acta2/Col12a1 gene expression (Fig. S4). These data highlight a striking clustering homology between the mammary tumour and distant metastasis.

### M-cluster in lung metastases was enriched in a hybrid mesenchymal/epithelial gene signature

To examine the relationship between the mesenchymal and luminal clusters at distant mets, we performed pseudo-temporal trajectory analysis on the integrated single cell transcriptomics between the GPx2KD tumour and paired lung mets using Monocle 3 [24]. Interestingly, cell trajectories and UMAP projections illustrate transitions between luminal and basal/mesenchymal cell lineages at the lung mets that followed a similar pattern as in the primary tumour (Fig. 3c, d). Namely, lung mets showed increased luminal (*Cdh1, Epcam, Cldn3/7*) and decreased mesenchymal (*Krt14, Krt17, Sox4/9*) gene expression with pseudotime distance between the mesenchymal and luminal clusters (Fig. 3e).

However, examination of each of the clusters for EMT marker expression in lung mets vs GPx2KD primary tumour, revealed upregulation of luminal (*Cdh1*, *Epcam, Cldn3/7*), basal/mesenchymal (*Krt17, Vim, Snai1/2, Twist1)* and (*Sox4, Jag1, Notch1)* genes in lung mets clusters, which was especially striking in mesenchymal (M) cluster 2 (Fig. 3f). Of note, the mesenchymal/epithelial (M/E) signature of cluster 2 in lung mets was contrasted with the highly mesenchymal signature of M-cluster 3 in primary tumour (Figs. 3f and S1A, B). These data implied M-cluster 2 in lung mets underwent a partial MET as compared to luminal clusters and M-cluster 3 in primary tumour which transited into partial EMT and full EMT respectively. These data were consistent with EMT and MET as key regulators of tumour invasion and metastasis respectively.

### HIF1α regulates metastasis via promotion of EMT dynamics at the primary tumour

We next tested whether GPx2 KD enhances metastasis via promotion of the hybrid E/M tumour phenotype. We showed that GPx2 KD in PyMT1 cells stimulates ROS/HIF1α signalling, causing vascular malfunction leading to hypoxia [9]. HIF1α was shown to promote intravasation via transcription of *Twist*, which activates N-cadherin and EMT [25]. HIF1α also promotes EMT via *Snai1*/*2* upregulation, thereby suppressing epithelial differentiation [26, 27]. In fact, GPx2 KD tumours showed HIF1α upregulation that was accompanied by increased SNAI1, TWIST, N-CAD and decreased E-CAD expression (see Fig. 2a, b), thus supporting effects of HIF1α on EMT.

**Fig. 4:**
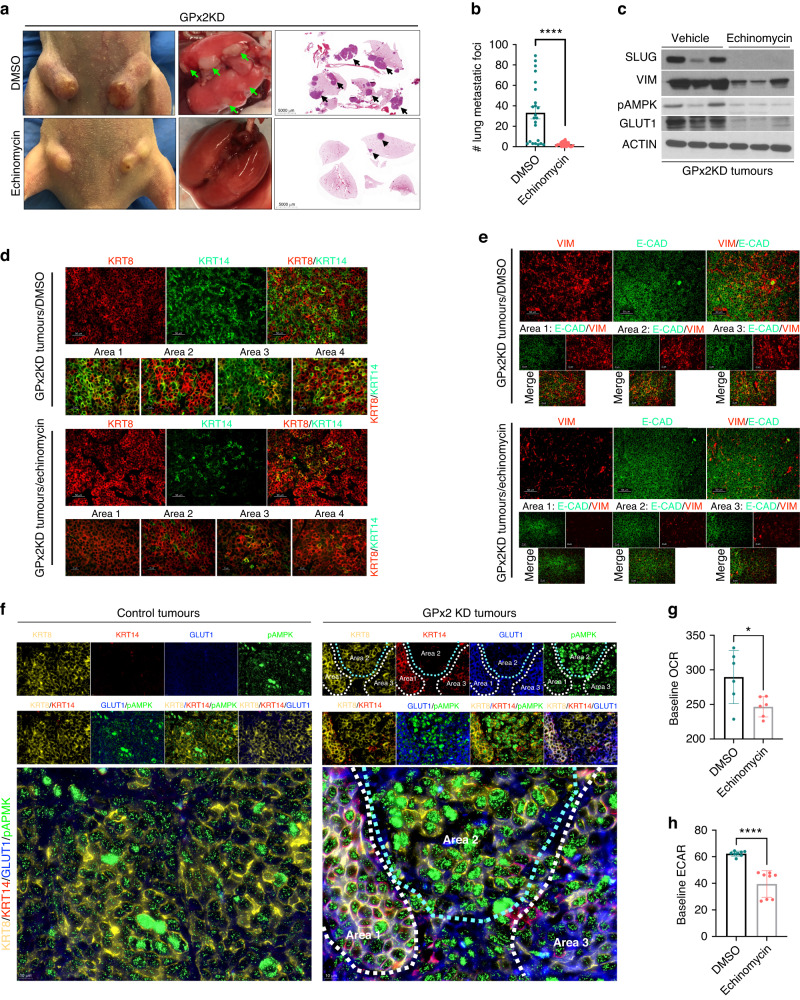
HIF1α regulates metastasis via promotion of hybrid EMT state and metabolic plasticity. **a** Athymic female mice (*n* = 10) bearing PyMT1/GPx2 KD tumours were treated post onset with daily i.p injection of vehicle (DMSO) or 10 μg/kg of echinomycin/DMSO for 21 days starting at 64 mm^3^ tumour volume. Representative tumours from both groups are shown (left boxes). Mice then underwent survival surgery to remove primary tumour and were left untreated for 1 week to recover. Treatment was then resumed daily for one more week as above. Four weeks later, mouse lungs were removed (second boxes), sectioned, H&E-stained and scanned; scans of whole lung lobes (third boxes) from both groups are shown (third boxes). **b** The number of lung foci from H&E stained sections are shown; Mean ± SEM; ****p < 0.0001. **c** GPx2 KD tumours from 3 independent mice that were treated with vehicle or echinomycin were immunoblotted for SLUG, VIM, pAMPK, GLUT1 vs ACTIN. **d**, **e** KRT8/KRT14 (**d**) or VIM and E-CAD (**e**) double-immunostained PyMT1/GPx2KD tumour sections treated with either or echinomycin from 3 mice each are shown. **f** Co-staining for four markers consisting of KRT8 (yellow), KRT14 (red), GLUT1 (blue), and pAMPK (green) shows individual and overlay staining. Robust quadruple staining was observed in GPx2KD tumours especially in Area 1 and also in part of Area 3, but not in Area 2 which was only KRT8 + /pAMPK+ (right panels). By contrast, control PyMT1 tumours were strongly KRT8 + /pAMPK+ (left panels). Staining was done in 3 mice (2 tumours each) in 3 sections from each tumour. **g**, **h** Bar graphs display baseline OCR and ECAR for GPx2 KD tumour cells treated with 5 nM echinomycin vs DMSO; normalised results are shown as Mean ± SEM; *p < 0.05, ****p < 0.0001.

To directly test the effects of HIF1α on metastasis in the PyMT1/GPx2 KD model, we used echinomycin, a drug that inhibits the interaction of HIF1α with DNA, thereby repressing HIF1α downstream gene transcription [28, 29]. Indeed, echinomycin suppressed VEGF-A and GLUT1, two bona fide HIF1α target genes, promoting angiogenesis and metabolism in the GPx2KD model [9]. To test the effects of echinomycin on spontaneous metastasis, GPx2KD tumour bearing mice were treated with echinomycin post-surgical excision of the primary tumour. This allowed us to avert mouse morbidity due to tumour burden and bypass poor drug perfusion due to abnormal vasculature [9]. One week post-surgical resection, mice were treated daily with intra-peritoneal injection of 10 μg/kg echinomycin or vehicle (DMSO) for 7 consecutive days, and then left untreated for 28 days to confirm effectiveness and durability of treatment. Remarkably, compared to vehicle-treated mice which produced a high load of metastatic nodules in the lungs, echinomycin-treated mice were nearly devoid of lung mets (Fig. 4a, b). Interestingly, echinomycin-treated tumours showed dramatic reduction in SLUG and VIM protein relative to vehicle-treated tumours (Fig. 4c). Interestingly, these tumours harboured significantly less KRT8/KRT14 or E-CAD/VIM positive areas than vehicle-treated GPx2KD tumours, due to mesenchymal gene repression (Fig. 4d, e). Of note, suppression of metastasis and EMT hybrid states by echinomycin in PyMT1/GPx2KD tumours was recapitulated by GPx2 OE or induction in PyMT2 tumours (Figs. 2 and S3), thus reiterating a strong connection between GPx2 KD, HIF1α, partial EMT, and metastasis.

### HIF1α coordinates phenotypic and metabolic plasticity at the primary tumour

HIF1α is a major driver of EMT and metabolism. Coupling gene regulation with cancer metabolism, others have demonstrated a stable hybrid metabolic state in metastatic breast cancer cells, consisting of oxidative phosphorylation (OXPHOS) and glycolysis [30, 31]. OXPHOS and glycolysis were associated with the activities of AMPK and HIF1α respectively. We have previously shown that GPx2 KD stimulates a shift from OXPHOS to aerobic glycolysis in all tumour cell clusters (cluster 0, 1, 2, 3, 4, 6), except for cluster 5 which used OXPHOS and glycolysis [9]. Immunostaining for phosphorylated-AMPK (pAMPK) and GLUT1 (downstream of HIF1α), showed that GPx2 KD tumours were enriched in cancer cells expressing both metabolic markers [9]. Interestingly, areas staining for pAMPK and GLUT1 were abolished in echinomycin-treated tumours (Fig. 4c), which is consistent with the notion that HIF1α may activate AMPK and glycolysis independently [32, 33].

To examine whether phenotypic and metabolic plasticity were interrelated, we co-stained tumours for luminal (KRT8; yellow) and basal (KRT14; red) markers, along with OXPHOS (pAMPK; green) and glycolysis (GLUT1; blue) markers. Remarkably, compared to control tumours which were KRT8 and pAMPK positive (Fig. 4f, left panels), GPx2 KD tumours contained KRT8/KRT14 positive areas that expressed pAMPK (in the cytosol) and GLUT1 (at the membrane) in same cells (Fig. 4f, right panels). Namely, compared to Area 2 which was pAMPK positive and GLUT1 negative (Fig. 4f, right panel), Area 1 and 3 showed reduced, yet positive expression of pAMPK, coinciding with strong expression of GLUT1, implying these areas were capable of using glycolysis, and to a lesser extent, also OXPHOS. We conclude that E/M subpopulations may leverage both glycolysis and oxidative respiration to survive under hypoxic or normoxic conditions.

Since HIF1α regulates pAMPK and GLUT1 expression, we tested whether echinomycin destabilises the E/M phenotype. Indeed, echinomycin-treated GPx2 KD tumours were negative for GLUT1, pAMPK and KRT14 but retained KRT8 expression relative to vehicle-treated GPx2 KD tumours which were positive for all four markers (Fig. S5A). Consistently, echinomycin-treated GPx2 KD tumour cells had significantly reduced levels of baseline oxygen consumption rate (OCR; OXPHOS) as well as of extracellular acidification rate (ECAR; glycolysis), relative to vehicle treated cells (Fig. 4g, h), further supporting pAMPK and GLUT1 as markers of dual metabolism. These data suggested that HIF1α regulates metastasis via promotion of E/M and M states.

Interestingly, inhibition of hybrid metabolism by echinomycin in GPx2KD cells was mimicked by GPx2 gain of function. Namely, PyMT2/GPx2OE tumours were negative for KRT8/KRT14/pAMPK/GLUT1 as compared to control tumours which were KRT8/pAMPK positive (Fig. S5B, C). Importantly, these data were recapitulated in human BC xenografts. Overexpression of GPx2 in the HER2-amplified JIMT1 cell line, which is GPx2 negative and brain-metastatic [34], suppressed mammary tumour growth (Fig. S6A, B), resulting in benign tumours with decreased VIM and increased E-CAD levels relative to control tumours (Fig. S6C). Of note, staining for E-CAD, VIM, GLUT1, pAMPK revealed co-expression of these markers in control JIMT-1 tumours (Fig. S6D, left panels; see arrows in bottom panels), indicative of hybrid metabolism in E/M cells. By contrast, GPx2 OE tumours were reduced in VIM/GLUT1 but increased in E-CAD/pAMPK expression as compared to control tumours (Fig. S6D**;** right panels). These findings underscored that GPx2 suppresses the E/M phenotype via inhibition of mesenchymal and glycolytic genes, resulting in E-CAD+/pAMPK+ tumours using OXPHOS.

### M-cluster cells colonising the lungs use OXPHOS as bioenergetic fuel

**Fig. 5:**
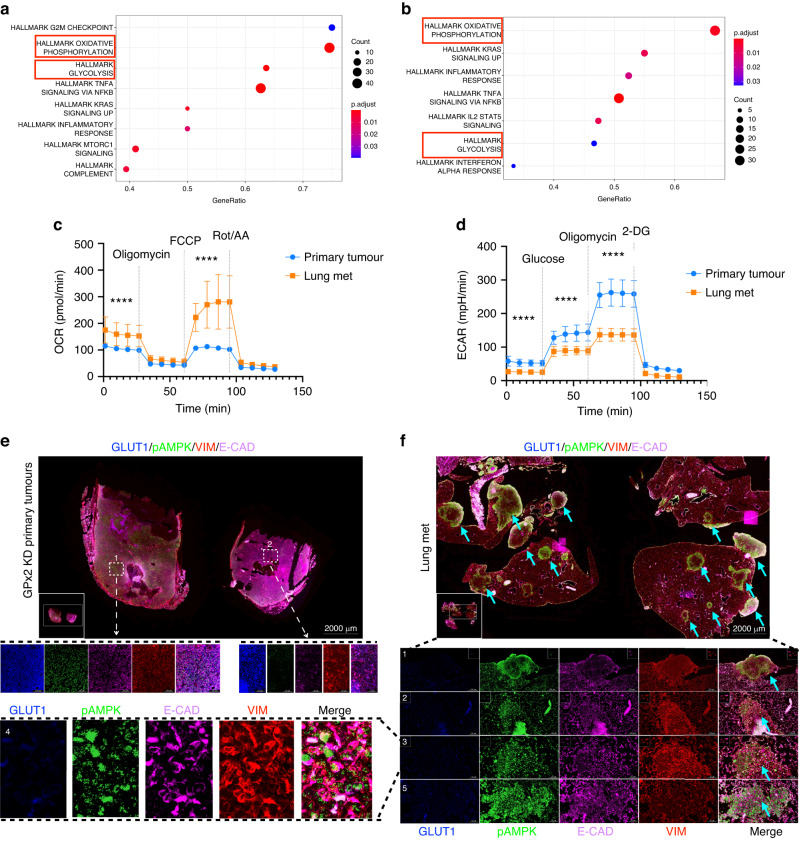
Lung metastases use OXPHOS as bioenergetic fuel. **a**, **b** Gene Set Enrichment Analysis (GSEA) revealed overrepresented pathways enriched in bulk lung mets (**a**) and in M-cluster (cluster 2) (**b**) indicating a clear predilection for OXPHOS over glycolysis in cluster 2. **c**, **d** PyMT1/GPx2 KD primary tumour and lung mets primary tumour cells were assayed for OCR and ECAR; normalised OCR data comparing both groups were derived for each of the mitochondrial respiration steps after 1 μM oligomycin, 1 μM FCCP, and 0.5 μM rotenone/antimycin treatment; normalised ECAR values were derived after sequential treatment with 20 mM glucose, 1 μM oligomycin, and 50 mM 2-DG; ****p < 0.0001. **e**, **f** Co-staining for GLUT1 (blue), pAMPK (green), VIM (red), and E-CAD (purple) shows individual and overlay staining on GPx2 KD primary tumour (*n* = 8) and paired lung mets (*n* = 4) from 4 mice. Representative images are shown.

Our findings underscored the phenotypic and metabolic plasticity of the primary tumour; however the metabolic status of lung mets remained unknown. To gain insights into signalling pathways preferentially activated in lung mets, we performed Gene Set Enrichment Analysis (GSEA) of bulk transcriptomics from lung mets vs primary GPx2KD tumour. As expected, OXPHOS, and to a lesser degree glycolysis, were enriched in lung mets (Fig. 5a). Moreover, GSEA of the differentially expressed genes (DEGs) between the mesenchymal clusters at the two sites revealed that OXPHOS was the top pathway activated in lung mets M-cluster 2 as compared to primary tumour M-cluster 3, which used glycolysis (Fig. 5b) [9]. These results corroborated findings in PDX models demonstrating a bioenergetic shift from glycolysis in mammary tumour to OXPHOS in lung mets [5]. In support of our GSEA-based findings, metabolic testing of carcinoma cells isolated from bulk primary tumour and lung mets, revealed dramatically higher levels of OCR and lower levels of ECAR in lung mets cells relative to primary tumour cells (Fig. 5c, d). These results were in contrast to the OCR^low^/ECAR^high^ profile of cancer cells derived from the GPx2KD primary tumour [9].

Further, examination of the metabolic profile in distant mets, revealed that lung mets were E-CAD/VIM/pAMPK positive and GLUT1 negative (Fig. 5f; area 1, 2, 3, highlighted in area 4). This was in contrast to GPx2KD mammary tumours which were E-CAD/VIM/pAMPK/GLUT1 (Fig. 5e; area 1) or VIM/GLUT1 positive (Fig. 5e; area 2). Of note, carcinoma areas expressing E-CAD/pAMPK, lacking VIM/GLUT1, were observed in macro-metastases, indicative of luminal differentiation driving MET (Fig. 5f; part of area 5). The shift to OXPHOS metabolism was *in sync* with the highly oxygenated environment in the lungs, which might be facilitated by the metabolic plasticity of E/M cells [5].

### p63 regulates the partial EMT and MET state in primary tumour and distant metastasis

**Fig. 6:**
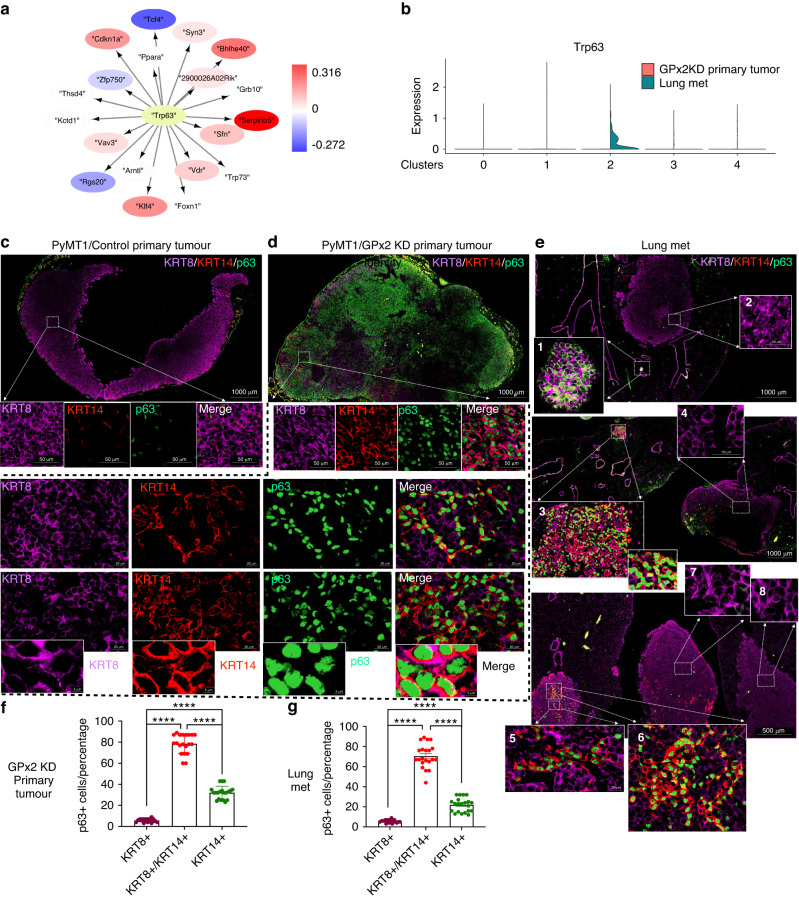
p63 regulates the partial EMT and MET state in primary tumour and distant metastases. **a** TF-TGs analysis using the Dorothea database shows abundant enrichment of *Trp63* target genes in lung mets. **b** Violin plots show p63 upregulation in cluster 2 relative to all other clusters when comparing lung mets to primary GPx2KD tumour. **c**–**e** Multiplex staining for KRT8 (purple), KRT14 (red), p63 (green) revealed that individual and overlay staining on control PyMT1 tumours (*n* = 8), PyMT1/GPx2 KD tumours (*n* = 8), and paired lung mets (4 independent lungs from 4 GPx2 KD tumour bearing mice). **f**, **g** Graphs display percentage of p63 positive cells that also expressed KRT8, KRT8/KRT14, or KRT14 in primary GPx2KD tumours (**f**) or lung mets (**g**); Mean ± SEM; ****p < 0.0001.

Next, we sought to elucidate the transcription factors (TFs) regulating target genes (TGs) in metastasis. By inputting the differentially expressed genes (DEGs) between lung mets and GPx2 KD primary tumour into the Dorothea database [35], we uncovered a dramatic enrichment of *Trp63* target genes in lung mets (Fig. 6a). Interestingly, *Trp63* mRNA was only expressed in M-cluster 2 but was absent from the luminal clusters in lung mets (Fig. 6b). Since M-cluster 2 gained luminal gene expression relative to the M-cluster 3 in primary GPx2KD tumour, we hypothesised that *Trp63* coordinates the entry of the M-cluster in the lungs into partial MET. This idea was consistent with studies demonstrating that *Trp63* regulates the hybrid state in primary tumour [36, 37], and led us to speculate that *Trp63* regulates partial EMT and MET at the tumour and metastatic sites respectively.

To test this hypothesis, we examined the levels of p63 in PyMT1/GPx2KD tumours vs control PyMT1 tumours and assessed p63 expression relative to the hybrid state. We triple co-immunostained tumours for p63, KRT8 (luminal) and KRT14 (basal) markers. Indeed, p63 nuclear staining was dramatically enhanced in GPx2KD relative to control tumours (Fig. 6c, d). Moreover, p63 was mostly co-localised with KRT8 and KRT14 in GPx2KD tumours (Fig. 6d) and this pattern was recapitulated in distant mets (Fig. 6e). However, the co-localisation of KRT8/KRT14 with p63 was noted in micro-mets but not in macro-mets which turned KRT8 positive (Fig. 6e; area 1 as compared to area 2). These data were confirmed in several independent mouse lungs, illustrating a predominant co-localisation of p63 with KRT8/KRT14 than with KRT8 or KRT14 alone (Fig. 6e; area 3 vs area 4; areas 5 and 6 vs areas 7 and 8). Quantification of GPx2 KD tumours and paired lung mets confirmed a higher percentage of carcinoma cells co-expressing p63 with KRT8/KRT14 than with KRT8 or KRT4 at both sites (Fig. 6f, g). These data implied that p63 upregulation by GPx2KD regulates the hybrid EMT state, thereby generating partial EMT and MET in primary and distant sites.

### HIF1α promotes p63 expression in vivo and in vitro

**Fig. 7:**
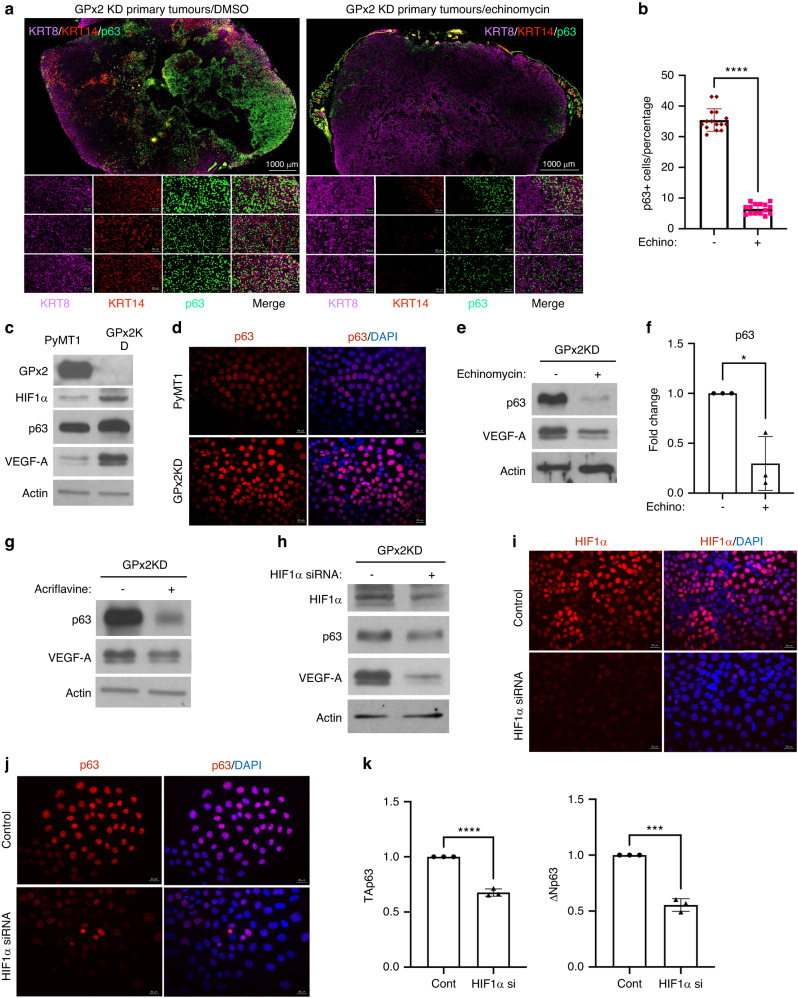
HIF1α regulates the partial EMT and MET state via p63 upregulation. **a** Co-staining of KRT8, KRT14, p63 showing individual and overlay staining in GPx2 KD tumours treated with DMSO (left panels) or echinomycin (*n* = 6) from 3 mice each. **b** Bar graphs display *p63* expression levels in GPx2 KD tumours that were treated with echinomycin vs DMSO (*n* = 6 each); ****p < 0.0001. **c** Western blots of GPx2, HIF1α, p63, VEGF-A vs Actin in PyMT1/GPx2 KD and control PyMT1 cells. **d** p63 IF staining of control and GPx2 KD cells showing upregulation/nuclear localisation of p63 in GPx2KD relative cells (bottom). **e**, **f** Western blots of p63 and VEGF-A vs Actin in GPx2 KD cells treated with DMSO or echinomycin (**e**); graphs show relative densitometry of p63 protein in 3 replicas (**f**); Mean ± SEM; *p < 0.05. **g**, **h** Western blots of p63 and VEGF-A vs Actin in GPx2 KD cells that were treated −/+ Acriflavine (**g**); or −/+ HIF1α siRNA (**h**). **i**, **j** IF staining of HIF1α (**i**) and p63 (**j**) on GPx2 KD/control cells vs GPx2 KD/HIF1α siRNA cells. **k** Graphs show TA and ΔN p63 isoform RNA levels in control cells vs siRNA treated GPx2KD cells; Mean ± SEM; ***p < 0.001, ****p < 0.0001.

Our data showed that HIF1α inhibition by echinomycin suppressed lung metastasis (Fig. 4a, b). To test whether GPx2KD regulates the hybrid state via p63 upregulation due to HIF1α signalling, we tested whether echinomycin suppressed the E/M phenotype. Indeed, echinomycin-treated GPx2 KD tumours showed dramatic reductions in p63 and KRT14 levels, resulting in KRT8-enriched tumours (Fig. 7a, b), indicative of luminal differentiation. Thus, our data implied that HIF1α regulates the E/M phenotype via upregulation of mesenchymal genes; however the connection between HIF1α and p63 in regulating the E/M tumour state remained unclear.

Interestingly, HIF1α upregulation in PyMT1/GPx2 KD cells was accompanied by upregulation of p63 and VEGF-A, a bona-fide HIF1α target gene (Fig. 7c). Of note, increases in p63 were more striking when comparing nuclear-localised p63 in GPx2KD cells vs control cells (Fig. 7d). To examine the link between HIF1α and p63, we used two independent HIF1α inhibitors, echinomycin and acriflavine [38], as well as HIF1α siRNA. VEGF-A was used to control for the activity of the HIF1α inhibitors as these do not interfere with HIF1α expression but with transcriptional activation [38]. Indeed, treatment of PyMT1/GPx2KD cells with echinomycin (5 nM) or acriflavine (5μM) for 18 h, caused a dramatic reduction in VEGF-A and p63 expression (Fig. 7e–g). Furthermore, HIF1α drug inhibition was mimicked by HIF1α siRNA in PyMT1/GPx2KD cells, causing significantly reduced p63 protein (Fig. 7h–j). Evaluation of the p63 isoforms regulated by HIF1α showed a significant reduction in both TA and ΔN p63 isoforms in HIF1α siRNA knockdown cells (Fig. 7k). These data demonstrate a close relationship between p63 and HIF1α in regulating the hybrid state, although the exact mechanism warrants further investigation.

## Discussion

Our findings highlight the far-reaching effects of GPx2 dysregulation on ROS/HIF1α signalling underlying EMT and metastasis. Our study provides novel insights into the diverse mammary tumour subpopulations and transcriptional regulators driving phenotypic and metabolic plasticity.

Single cell transcriptomics unravelled dramatic effects of GPx2 KD on EMT, resulting in tumour subpopulations drifting through an EMT continuum. Notably, GPx2 KD caused de novo induction of mesenchymal genes in luminal clusters as well as enhanced the mesenchymal signature of cluster 3. By arbitrarily placing cluster 3 at the origin of the lineage trajectory as the mesenchymal transcriptional node, we uncovered closest proximity between cluster 5 and 3 in pseudotime distance, implying cluster 5 might be an early hybrid cluster. This is consistent with the hybrid metabolic status of cluster 5, underscoring a link between phenotypic and metabolic adaptation [9]. In fact, studies in skin, breast and pancreatic cancers unravelled early and late hybrid EMT clusters, with the early clusters in breast exhibiting robust tumour initiating and metastatic potential [15, 16].

These studies argue against mesenchymal tumour cells as the pivotal drivers of metastasis, which precludes mesenchymal cluster 3 from initiating this process [39]. In fact, others have shown that E/M cells were tumour-initiating and metastatic relative to E or M cells [13, 14]. We speculate that the M subpopulation (cluster 3) lies at the apex of the hierarchy, giving rise to bipotent progenitors that differentiate into an E or M state. Alternatively, E carcinoma cells may convert into E/M and then M trough a stepwise transition process. Interestingly, we showed that luminal clusters in primary tumour gained mesenchymal genes, whereas the mesenchymal cluster 2 in the lungs acquired *de-novo* luminal gene expression. Hence, we speculate that E/M or M/E cells may be bipotent progenitors that differentiate into E or M clusters in response to TME cues [40].

HIF1α appears to play a pivotal role in EMT dynamics by promoting mesenchymal gene expression in luminal as well as in mesenchymal clusters at the primary tumour, thereby generating hybrid E/M and extra-M subpopulations. HIF1α is known to activate *Snail1/2*, which in turn represses *Cdh1* among other epithelial genes, causing E to M switch. However, HIF1α may independently activate mesenchymal genes such as *Vim*, *Twist2*, and *Cdh2* in luminal clusters [25, 41], thus generating an E/M phenotype. Of note, *Twist*, and not *Vim*, was upregulated in cluster 2 in lung mets relative to cluster 3 in primary tumour, thus supporting other findings that N-CAD (downstream of TWIST) was more critical than VIM in driving the metastatic process [42].

Importantly, our data highlight the notion that transcriptional regulation of partial EMT or partial MET occurs via p63, which corroborates findings showing tight transcriptional control of the hybrid state by p63 [37]. Indeed, we showed that hybrid cells in GPx2 KD primary tumour and lung mets were enriched in p63, thus supporting a critical role in the onset of hybrid populations at both sites. Remarkably, p63 expression was enriched in micro-, but not, in macro- metastases, suggesting p63 regulates partial MET that progress into full MET upon proliferation/differentiation of metastatic clusters. Others have shown that p63 was co-localised with a partial EMT programme at the leading edge of head and neck tumours [43], and was also enriched in collectively migrating cells or CTCs containing E/M cells [44, 45]. Of note, p63 promotes mammary gland maturation via transactivation of NRG1 in the luminal layer by p63 in the basal layer, resulting in ERBB4/STAT5 signalling driving luminal differentiation [36], a process which remains to be validated in vivo in the tumour context.

Interestingly, GPx2 KD appears to regulate p63 via HIF1α, leading to the onset of partial EMT. Of note, p63 KD in SUM159 basal-like BC cells suppressed lung colonisation, suggesting a critical role for p63 in metastasis [37, 46]. Indeed, we showed that echinomycin inhibits p63 and E/M marker expression while eradicating metastasis, thus suggesting p63 promotes tumour spread via the EMT hybrid state. Whether this regulation is direct or indirect remains to be validated. In favour of an indirect mechanism, we posit that HIF1α perturbation, which inhibits mesenchymal gene expression, destabilises p63 and hence the E/M state. Moreover, GPx2 KD may regulate this process via oxidation/inactivation of tyrosine phosphatases by ROS [47], which results in EGFR/STAT3 signalling, leading to p63 gene transcription [48, 49]. Otherwise, HIF1α may activate Wnt signalling in hypoxic niches, thereby upregulating p63, a well-documented Wnt target gene [47, 49, 50].

Other than promoting EMT hybrid states, HIF1α regulates tumour metabolism, and these processes might be tightly interwoven [30, 51]. In fact, echinomycin suppressed VIM, SNAIL, pAMPK and GLUT1 implying far-reaching effects of HIF1α on EMT and metabolism. Indeed, echinomycin suppressed OXPHOS (OCR) and glycolysis (ECAR) as well as pAMPK and GLUT1 which are markers of these metabolic processes. These data were consistent with activation of AMPK by HIF1α, which in turn stimulates fatty acid oxidation (FAO) and hence OXPHOS [52–54]. By contrast to the clusters in the primary tumour which mostly utilised glycolysis, lung mets clusters, especially cluster 2, used OXPOS likely as an adaptive response to the oxygenated environment in the lungs [5, 6, 51, 55]. The relationship between EMT and metabolism remains unclear despite reports of mutual cross regulation [56]. A study of 180 cancer cell datasets showed that the mesenchymal phenotype positively correlates with glycolysis and inhibition of OXPHOS, whereas the epithelial phenotype favours OXPHOS/FAO [57, 58]. Hence, it is not surprising that cells transiting through an hybrid EMT state may use OXPHOS and glycolysis to allow for metabolic flexibility in an evolving TME. Thus, cross regulation between EMT and metabolism warrants further investigation.

The plasticity of the hybrid EMT state remains debatable. Studies using gene regulatory networks from lung cancer single cell datasets, were able to map teams that were “strong” yielding stable phenotypes such as the epithelial or mesenchymal state, or “weak” yielding unstable or plastic phenotype such as the E/M hybrid state [13]. We annotated published team factors [57] in our single cell datasets but were unable of detecting any of the candidate team factors across our clusters. This is however not surprising in light of the high sparsity/drop out rate that is associated with the single cell RNA technology. Team players set aside, empirical evidence suggests that the hybrid EMT state may reside in a transiently stable state [40, 59]. In fact, E/M cells which were isolated from basal-like BC cell lines based on CD104 and CD44 expression, were stable in culture over several passages [13]. Moreover, E/M and M cells were shown to be dependent on Snail and Zeb-1 expression respectively. Hence, trapping carcinoma cells in the E/M state, was achieved by Snail overexpression combined with Zeb-1 knockdown [13]. Since Snail is negatively regulated by Mir 34, and Zeb-1 by Mir 200, we surmise that regulatory networks causing differential expression of these microRNAs contributes to the establishment of the hybrid EMT state [57, 58]

Altogether, our findings illustrate the complex dynamics regulating tumour progression and metastasis with emphasis on GPx2KD/HIF1α/p63 signalling driving the onset of tumour cell subpopulations undergoing phenotypic and metabolic plasticity. Such novel insights have the potential to inform therapeutic strategies for metastatic breast cancer.

### Supplementary information

Figure S1

Figure S2

Figure S3

Figure S4

Figure S5

Figure S6

Supplemental Figure legends

## Data Availability

The single-cell RNA sequencing data of primary tumour and lung metastases reported in this manuscript are available at https://www.ncbi.nlm.nih.gov/geo with accession numbers GSE152368 and GSE215394. Coding analyses for single-cell RNA sequencing data are available in GitHub with the URL accession link https://github.com/Malindrie/Breast-cancer-scRNA-seq-analysis. All reagents and resources are available from rachel.hazan@einsteinmed.edu.
