# Supplementary figures and images for: Redox signalling regulates breast cancer metastasis via phenotypic and metabolic reprogramming due to p63 activation by HIF1α
Source: Br J Cancer. 2024 Jan 18;130(6):908–24. doi: 10.1038/s41416-023-02522-5 (PMC10951347; doi:10.1038/s41416-023-02522-5)

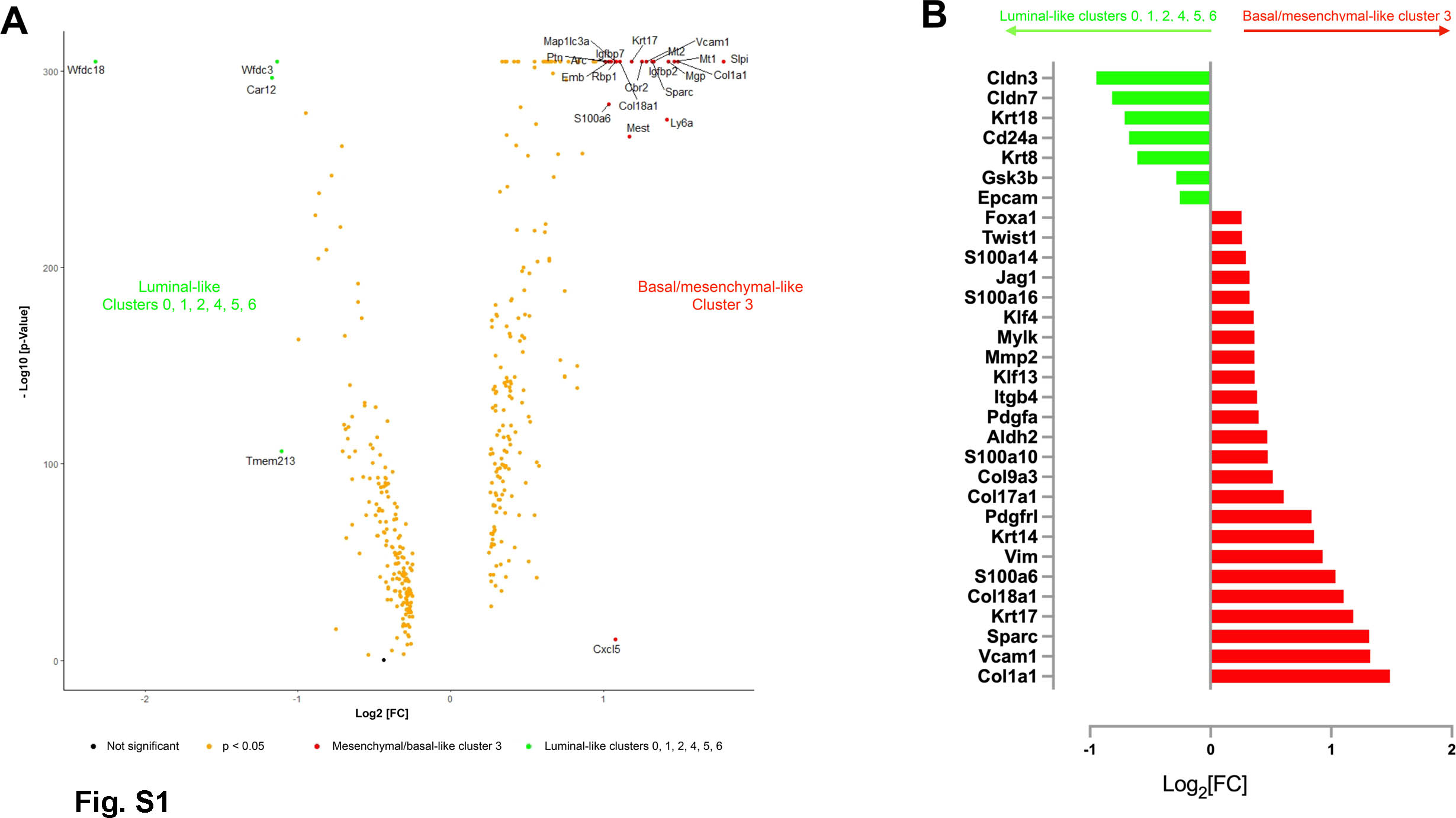

Supplement: Supplementary file 1 — Figure S1 [file 41416_2023_2522_MOESM1_ESM.jpg]

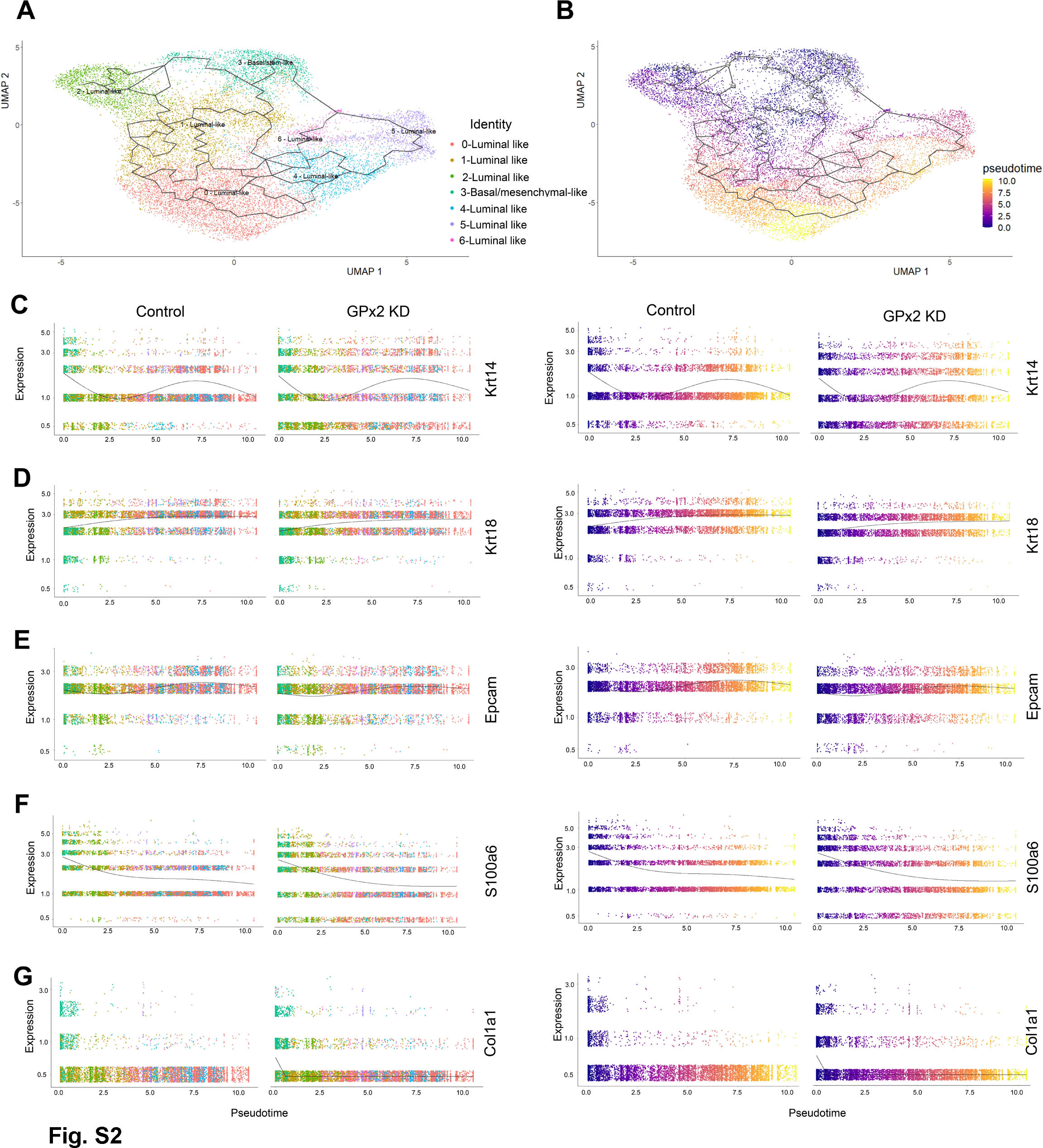

Supplement: Supplementary file 2 — Figure S2 [file 41416_2023_2522_MOESM2_ESM.jpg]

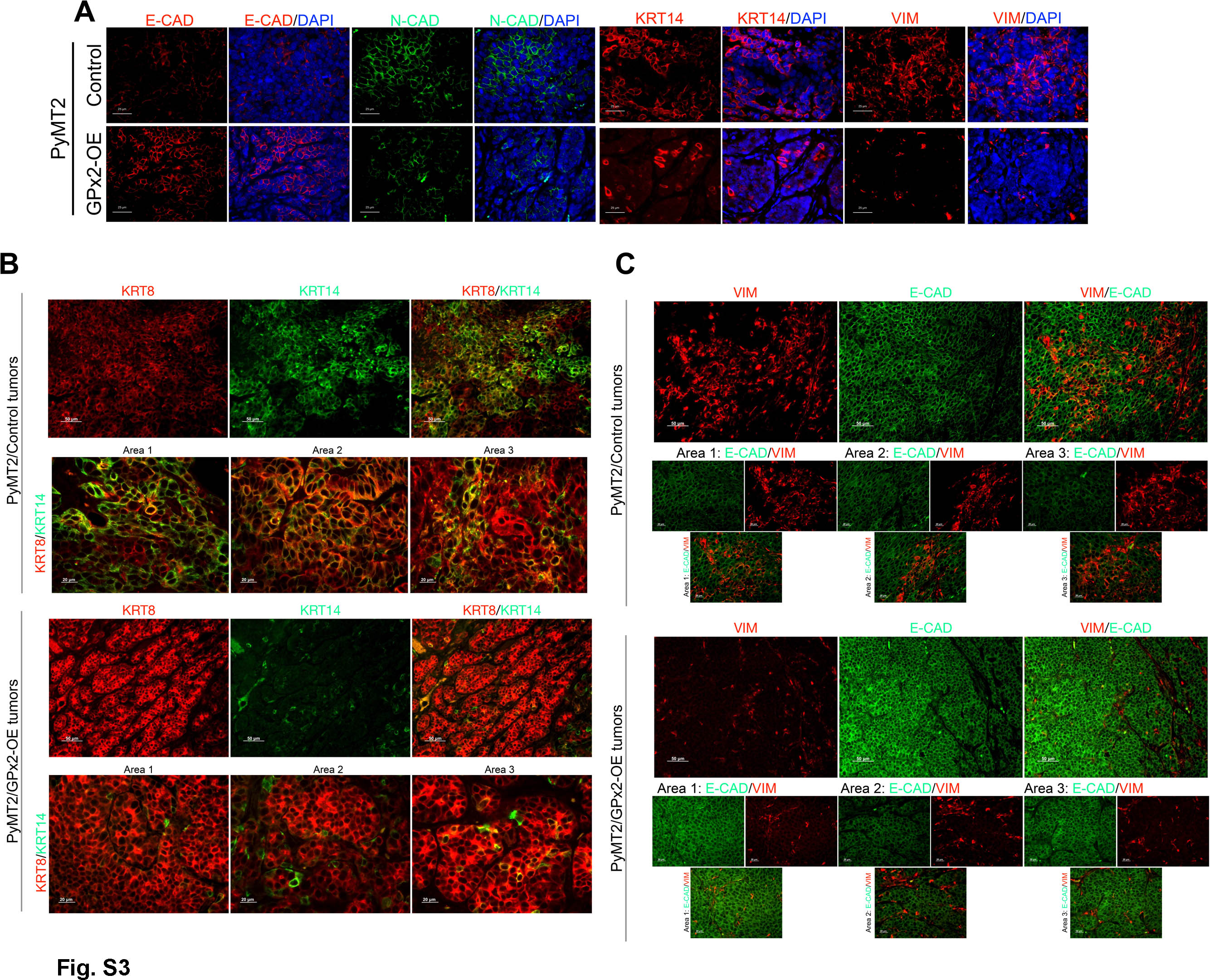

Supplement: Supplementary file 3 — Figure S3 [file 41416_2023_2522_MOESM3_ESM.jpg]

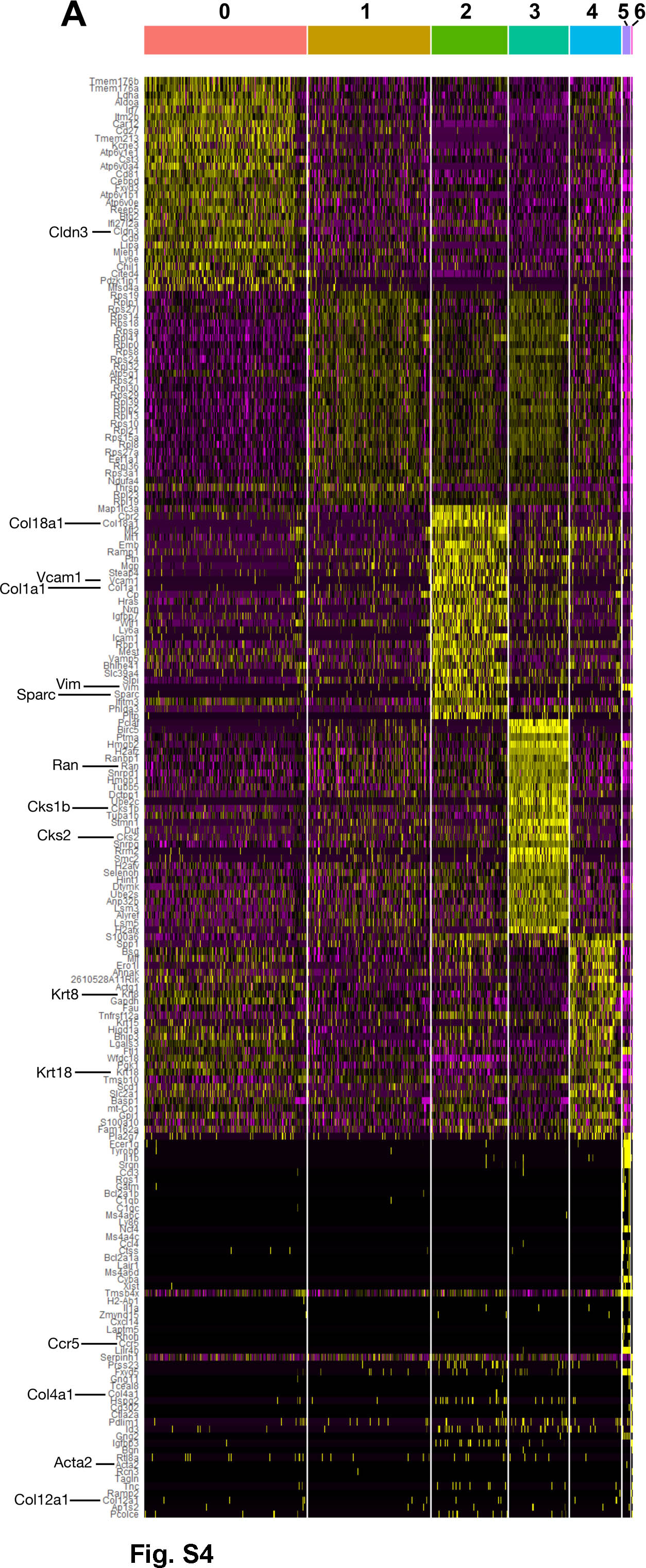

Supplement: Supplementary file 4 — Figure S4 [file 41416_2023_2522_MOESM4_ESM.jpg]

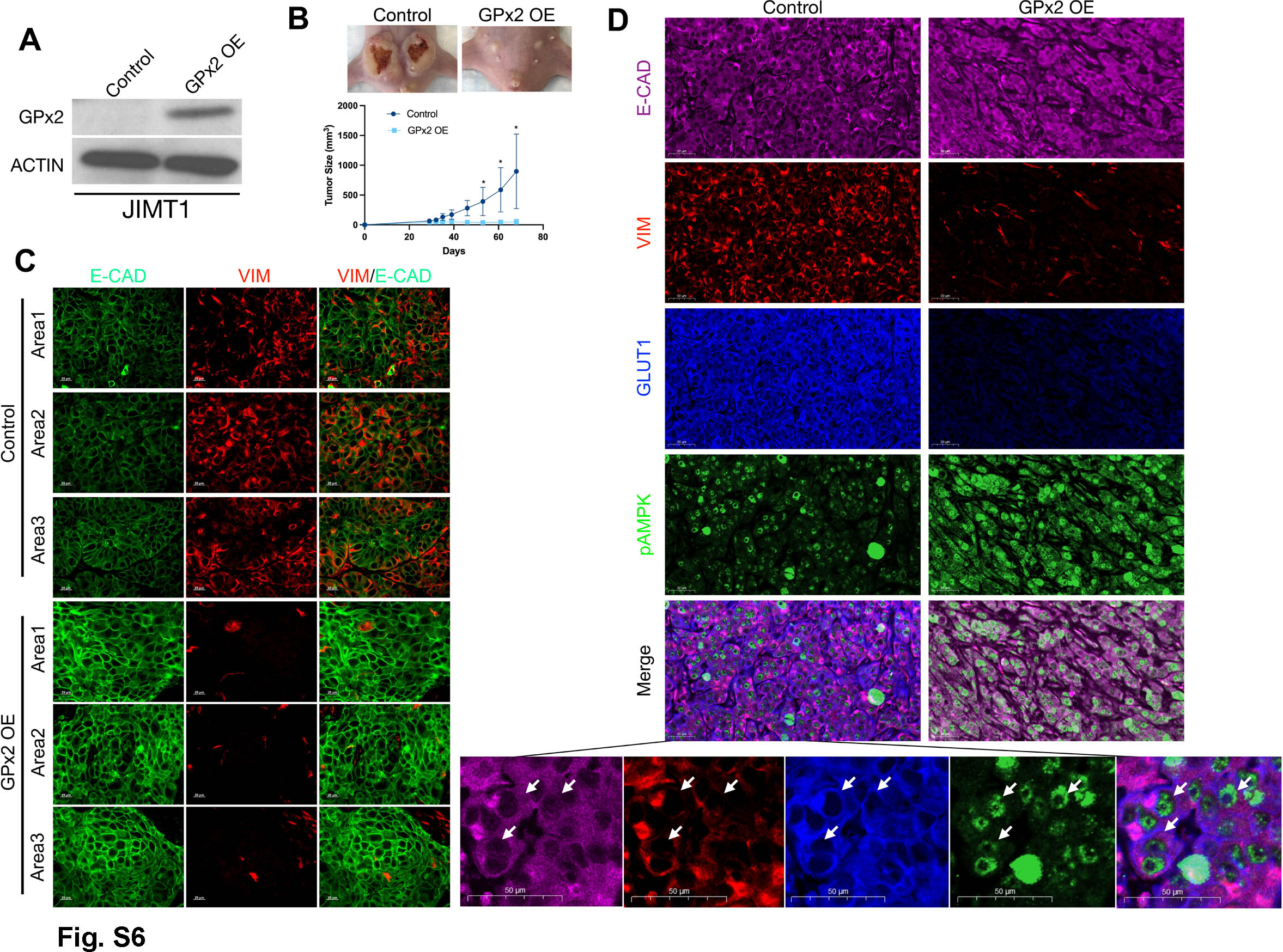

Supplement: Supplementary file 6 — Figure S6 [file 41416_2023_2522_MOESM6_ESM.jpg]
