# Supplementary material for: Redox signalling regulates breast cancer metastasis via phenotypic and metabolic reprogramming due to p63 activation by HIF1α
Source: Br J Cancer. 2024 Jan 18;130(6):908–24. doi: 10.1038/s41416-023-02522-5 (PMC10951347; doi:10.1038/s41416-023-02522-5)
Supplement: Supplementary file 7 — Supplemental Figure legends [file 41416_2023_2522_MOESM7_ESM.pdf]

Supplemental figures

Fig. S1. scRNAseq reveals basal/mesenchymal cluster 3 is enriched in an EMT gene signature. (**A**) Volcano plot shows the differentially expressed genes between cluster 3 (cell number: 1666) and clusters 0, 1, 2, 4, 5, 6 (cell number: 13433). X-axis is a Log transformation of mRNA fold change at base 2. Y-axis is a negative Log transformation of p-Value at base 10. Yellow dot indicates significantly expressed genes ($p < 0.05$); Green dot highlights top genes with log2 [FC] less than -1 and -log10 [p-Value] greater than 100 that are significantly upregulated in luminal-like clusters 0, 1, 2, 4, 5, 6. Red dot highlights top genes with log2 [FC] greater than 1 and -log10 [p-Value] greater than 100 that are significantly upregulated in basal/mesenchymal-like cluster 3. (**B**) Waterfall plot shows log2[FC] values for the differentially and significantly expressed EMT and stem-like genes in cluster 3 *vs* clusters 0, 1, 2, 4, 5, 6 ($p < 0.05$).

Fig. S2. Cell trajectory analysis highlights a transcriptional EMT continuum across clusters

(**A**) UMAP projection of six luminal-like cluster and one basal/mesenchymal-like cluster from PyMT1 control and PyMT1/GPx2 KD tumors; the bifurcated line indicates a branched trajectory from the root of the path to one of the terminal nodes. (**B**) Pseudotime time trajectories projected onto UMAP in (A); colors reflects pseudotime distance between the clusters with cluster 3 representing the mesenchymal transcriptional node, located at $t = 0$. (**C-G**) Gene expression levels of luminal/epithelial (*Krt18*, *Epcam*) and basal/mesenchymal (*Krt14, S100a6, Col1a1*) genes in individual cells across all clusters over pseudotime in PyMT1/GPx2 KD *vs* control PyMT1 tumor are shown; colors (left two columns) reflect individual clusters in UMAP projection over pseudotime distance, and colors (right two columns) display pseudotime distance (basal/mesenchymal cluster 3 located at $t = 0$).

Fig. S3. Exogenous expression of GPx2 in PyMT2 cells inhibits the EMT continuum

(**A**) Immunofluorescent staining for E-CAD, N-CAD, KRT14, and VIM in PyMT2 control tumors (n=6) and PyMT2/GPx2-OE tumors (n=6) from 3 mice each. Three random sections from each tumor were

immunostained. Representative images are shown. (**B-C**) Co-immunostaining of KRT8 with KRT14 (B), or VIM with E-CAD (C) in PyMT2 control tumors and PyMT2/GPx2-OE tumors from 3 mice each.

Fig. S4. Heatmap of unsupervised clustering of top 30 differentially expressed genes between lung mets and primary GPx2KD tumor using integrated transcriptomics. Four luminal clusters (cluster 0, 1, 3, 4) expressing (*Cldn3, Krt8, and Krt18*); cluster 3 was enriched in proliferative genes (*Ran, Cks1b, and Cks2*). Cluster 2 was basal/mesenchymal-like (*Col18a1, Vcam1, Col1a1, Vim, Sparc*); cluster 5: macrophage-like (Ccr5, Col4a1), and cluster 6: fibroblast-like (Acta2, Col12a1).

Fig. S5. GPx2 OE or HIF1α inhibition suppresses EMT dynamics and metabolic plasticity

(**A**) Co-staining for KRT8/KRT18/GLUT1/pAMPK in GPx2 KD tumors (n=6) that were treated with DMSO (left) or echinomycin (right) from 3 mice is shown in representative sections. (**B-C**) Co-staining the four above markers in PyMT2 control vs PyMT2/GPx2OE tumors (B) and in PyMT2/dCas9 vs PyMT2/GPx2-gRNA2 tumors (C); (n=6) from 3 mice each.

Fig. S6. GPx2 OE in human BC JIMT1 cells suppresses tumor growth and EMT dynamics

(**A**) Immunoblotting analysis of GPx2 vs ACTIN in JIMT1 control and GPx2 OE cell lysates. (**B**) JIMT1 control and GPx2 OE cells (1 x 10^6) were bilaterally injected into mammary fat pads of female athymic nude mice (n=3 each group); representative images of tumor growth at 68 days post onset are shown (upper panels); Mean ±SEM; *$p < 0.05$. (**C**) Immunofluorescent co-staining for VIM and E-CAD in GPx2 OE vs control JIMT1 tumors (n=6) from 3 mice each. (**D**) Co-staining for E-CAD/VIM/GLUT/pAMPK in GPx2 OE vs control JIMT1 tumors (n=6).
